# TM4SF5-mediated KEAP1 Regulation in Hepatocytes Irrelevant to NRF2 Expression and Activity Promotes Oxidative Stress and Inflammation to Develop Metabolic Dysfunction-Associated Steatotic Liver Disease

**DOI:** 10.7150/ijbs.126251

**Published:** 2026-01-01

**Authors:** Eun-Ae Shin, Haesong Lee, Kyung-hee Pyo, Wonsik Kim, Soyeon Kim, Jae-Ho Lee, Seo Hee Jin, Eunmi Kim, Soo-Min Byeon, Dong Joo Kim, Young Jun Cho, Tae Won Kim, Minjae Ohn, Hyojung Lee, Jeongwon Lee, Jinwook Jeong, Doojin Kim, Jie Zheng, Han Ah Lee, Hwi Young Kim, Young-Joon Surh, Jung Weon Lee

**Affiliations:** 1 Department of Pharmacy, College of Pharmacy, Seoul National University, Seoul 08826, Republic of Korea.; 2 Research Institute of Pharmaceutical Sciences, College of Pharmacy, Seoul National University, Seoul 08826, Republic of Korea.; 3 Department of Surgery, Gachon University Gil Medical Center, Gachon University School of Medicine, Incheon 21565, Republic of Korea.; 4 Department of Internal Medicine, Chung-Ang University College of Medicine, Seoul 06974, Republic of Korea.; 5 Department of Internal Medicine, Ewha Womans University College of Medicine, Division of Gastroenterology and Hepatology, Ewha Womans University Mokdong Hospital, Seoul 07985, Republic of Korea.

**Keywords:** lipid toxicity, KEAP1, metabolic dysfunction-associated steatohepatitis, reactive oxygen species, tetraspanin

## Abstract

Metabolic dysfunction-associated steatohepatitis (MASH)-associated fibrosis involves inflammation accompanied by reactive oxygen species (ROS), in addition to abnormal lipid metabolism and extracellular matrix (ECM) deposition. ROS levels are regulated by the NRF2-KEAP1 pathway. Transmembrane 4 L six family member 5 (TM4SF5) is implicated in metabolic dysfunction-associated steatotic liver disease (MASLD). However, it remains unknown how hepatocyte TM4SF5 modulates abnormal lipid and ROS accumulations during MASLD development. Here we assessed the influence of TM4SF5 on NRF2-KEAP1 pathway utilizing various *in vitro* and *in vivo* MASLD models. Our results indicate that hepatocyte TM4SF5 downregulates KEAP1 in physiological states and stabilizes KEAP1 in pathological conditions, without altering NRF2 expression. However, TM4SF5-dependent stabilization of KEAP1 was not observed in *Tm4sf5*^-/-^ KO mice. At least the cytosolic TM4SF5 C-terminus could bind to KEAP1 for proteosomal degradation. TM4SF5-driven biphasic KEAP1 regulation was associated with increased CD36 levels in normal livers, whereas in hyperlipidemic states, it contributed to oxidative stress and hepatic inflammation. Genetically engineered mice with altered *Tm4sf5* and *Nrf2* displayed TM4SF5-induced MASLD phenotypes characterized by elevated Keap1, regardless of Nrf2 expression or activity. These findings were more obvious than for mice with Nrf2 mutation alone. Notably, suppression of Keap1 alone nullified the MASLD-promoting effects of TM4SF5. Taken together, these data demonstrate that TM4SF5 can modulate KEAP1 independently of NRF2, identifying TM4SF5-mediated KEAP1 stabilization as a potential therapeutic target for MASLD.

## Introduction

Chronic liver injury may result from abnormal metabolic dysfunctions and an inflammatory environment characterized by the excessive presence of reactive oxygen species (ROS), which leads to a persistent pathological state that predisposes individuals to metabolic dysfunction-associated steatohepatitis (MASH) and fibrogenesis in the liver 1. Additionally, hepatocyte damage triggered by lipid toxicity resulting from abnormal lipid metabolism and accumulation, induces ROS generation, inflammation, fibrosis, and metabolic dysfunction-associated steatotic liver disease (MASLD), which affects approximately 30% of the global population and ultimately contributes to hepatocellular carcinogenesis 2, 3. In the setting of chronic liver diseases, metabolic and inflammatory mediators, along with components of the immune system, play critical roles in the dysregulation of nutrient utilization and metabolic processes within an inflammatory milieu. Within such an environment, elevated levels of reactive oxygen species (ROS) can promote MASLD, while antioxidant defense mechanisms such as the NRF2-KEAP1 pathway have been shown to play a protective role in limiting the development of MASLD 4. Prolonged liver injury associated with ROS may facilitate the progression of pathological changes that include hepatic lipid accumulation (steatosis), inflammation (steatohepatitis), and excessive deposition of extracellular matrix (ECM) (fibrosis), which together drive the development of MASLD 5. The accumulation of excessive ECM can induce abnormal cellular proliferation and result in cirrhosis associated with scar tissue formation, ultimately progressing to liver failure and hepatocellular carcinoma (HCC) 6. Nevertheless, the specific molecular mechanisms that govern the transition from normal liver physiology to HCC via MASLD remain complex and not fully elucidated. The identification of biomarkers indicative of the progression from MASH to HCC may facilitate the development of effective therapeutic and clinical interventions 7. Furthermore, environmental cues, including immunological mediators, effectors, and ROS, are known to facilitate the advancement of pathological conditions 8, 9. Multiple cellular processes, such as gene expression, proliferation, and migration, are highly influenced by the extracellular environment, which comprises ECM proteins, ROS, and soluble factors like cytokines and chemokines 8. As a result, membrane receptors or proteins are essential in mediating bidirectional communication between the intracellular and extracellular compartments throughout the development and progression of chronic liver disease.

Transmembrane 4 L six family member 5 (TM4SF5) is a transmembrane glycoprotein that belongs to the transmembrane 4 L six family, which is a subgroup of the tetraspan(in) protein family. It demonstrates high expression in diverse cancers, including HCC 10. In hepatocytes, TM4SF5 expression can be upregulated by TGFβ1-mediated Smad signaling and EGFR activation during the progression of fibrosis in animal liver models 11. TM4SF5 further contributes to hepatic steatosis and steatohepatitis in mouse models exposed to high-fat or high-carbohydrate diets 12, 13. In addition to its function in MASLD, hepatocyte-specific overexpression of *Alb-*Tm4sf5 (where the albumin promoter is linked with the mouse *Tm4sf5* gene) predisposes to liver cancer development at advanced age, or following chemical challenge or xenograft implantation 14. Nevertheless, even across these *in vitro* and/or *in vivo* TM4SF5-dependent liver disease models, the potential involvement of reactive oxygen species (ROS) in TM4SF5-driven MASLD or liver cancer remains uninvestigated. Although transgenic mice with hepatocyte-specific TM4SF5 overexpression exhibit varying degrees of liver steatosis, fibrosis, and tumorigenesis depending on age and disease susceptibility, the specific roles of ROS modulation via the NRF2-KEAP1 pathway in mediating TM4SF5 actions have not been clarified.

The signaling pathway involving the transcription factor NF-E2-related factor 2 (NRF2) and Kelch-like ECH-associated protein 1 (KEAP1) is recognized as a primary regulator of cellular defense mechanisms against environmental stresses through modulation of ROS 15. NRF2 triggers the upregulation of antioxidant enzymes that facilitate ROS detoxification. In contrast, KEAP1 governs NRF2 activity and acts as a molecular sensor for oxidative or electrophilic stress 16. Under non-stress conditions, the ubiquitin ligase CUL3-KEAP1 complex mediates the ubiquitination of NRF2, promoting its degradation via the proteasome. When cells are exposed to stress, NRF2 escapes degradation and accumulates in the nucleus, whereas the levels and localization of KEAP1 and CUL3 remain constant. Once in the nucleus, NRF2 forms a heterodimer with sMAF (small MAF proteins), enabling the complex to bind the CNC-sMAF binding element (CsMBE) to initiate transcription of target antioxidant genes 15. Although the majority of research indicates an inverse correlation between NRF2 and KEAP1 in modulating inflammation, some studies have reported a lack of association or independent function of these proteins 17, 18.

In this study, we explored whether ROS or the NRF2-KEAP1 pathway might contribute to TM4SF5-mediated development of MASLD features, utilizing both *in vitro* cellular and *in vivo* animal models. Unexpectedly, our findings revealed that TM4SF5-mediated effects on MASLD development and progression predominantly involved KEAP1, while NRF2 was not implicated. These observations led us to propose that TM4SF5 may interact with KEAP1 independently of NRF2, ultimately promoting MASLD development. We demonstrated that TM4SF5 directly bound to and regulated KEAP1 and CD36, without significantly affecting NRF2 levels or its functions. These molecular alterations appeared sufficient to drive MASLD features, suggesting that targeting the TM4SF5-KEAP1 interaction could be a promising therapeutic strategy for MASLD.

## Materials and Methods

**Cell culture, transfection, and reagents:** Human hepatocellular carcinoma cell lines without TM4SF5 expression (SNU449 and SNU761) and cell lines expressing TM4SF5 (Huh7, HepG2, and Hep3B) were acquired from the Korean Cell Line Bank (Seoul National University, Seoul, Korea). The murine normal hepatocyte AML12 cell line, lacking TM4SF5, was obtained from the American Type Culture Collection (ATCC, Manassas, VA, USA). SNU449, SNU761, and Hep3B cell lines were cultured in RPMI-1640 medium (SH30027.01, Hyclone, Logan, UT, USA). Huh7 and HepG2 cell lines were cultured in DMEM (SH30243.01, Hyclone). Both culture media were supplemented with 10% FBS (F0600, GenDEPOT, Barker, TX, USA) and 1% penicillin/streptomycin (CA005, GenDEPOT). AML12 cells were cultured in DMEM/F12 (SH30023.01, Hyclone) supplemented with 10% FBS, 10 μg/ml insulin, 5.5 μg/ml transferrin, 6.7 ng/ml selenium (ITS, 41400045, Gibco, Waltham, MA, USA), and 40 ng/mL dexamethasone (265005, Sigma-Aldrich, St. Louis, MO, USA). All cultures were maintained at 37°C in a humidified atmosphere with 5% CO_2_. All cell lines were subcultured every 3-4 days at the recommended ratio, with routine mycoplasma testing to monitor contamination. For gene expression studies and viral vector production, cells were transfected with cDNA plasmids using polyethyleneimine (PEI, sc-360988, Santa Cruz Biotechnology). To generate tetracycline-inducible shRNA systems, MISSION^®^ shRNA constructs provided by Sigma-Aldrich were ligated into EZ-Tet-pLKO-vector (#85966 or #85973, Addgene). We utilized annealed oligonucleotides targeting KEAP1 (TRCN0000154656, TRCN0000154657, TRCN0000156676, and TRCN0000158081) and NRF2 (TRCN0000007558, TRCN0000007555, TRCN0000273494, and TRCN0000007556). Stable cell lines expressing Tet-shRNA were treated with 0.2 μg/ml doxycycline every 2 days to induce shRNA expression. The reagents employed in our *in vitro* studies included: actinomycin D (A9415, Sigma-Aldrich), 10 μM MG132 (M-1157, AG Scientific), chloroquine (C6628, Sigma-Aldrich), and 50 μg/ml cycloheximide (C4859, Sigma-Aldrich). To model MASH (or NASH) *in vitro*, cells were treated with BSA-palmitic acid conjugate solution (P0500 and A6003, Sigma-Aldrich), a lipid mixture (L0288, Sigma-Aldrich), cholesterol (Sigma-Aldrich), and free fatty acid solution (F7050, Sigma-Aldrich).

**Animals:** All mice were maintained in a pathogen-free facility under controlled humidity and temperature conditions. All animal experiments were complied with the ARRIVE guidelines. All animal experiments followed the guidelines outlined in the Seoul National University Laboratory Animal Maintenance Manual and received approval from the IRB of the Institute of Laboratory Animal Resources, Seoul National University (SNU181016-7-4, SNU-200706-4, SNU-220920-2, and SNU-230712-1-1). Tm4sf5 knockout (*Tm4sf5*^-/-^) and hepatocyte-specific Flag-tagged Tm4sf5 overexpressing transgenic (*Alb*-TG^Tm4sf5-Flag^) C57BL/6 mice were produced as previously described 12, 19. Nrf2^mut^ mice are deficient in the ability of Nrf2 to bind DNA due to a deletion in the CNC bZIP region (amino acids 452 to 560) 20. Nrf2^mut^ mice were generously provided by Yuet Wai Kan's laboratory. *Tm4sf5*^-/-^×Nrf2^mut^ and *Alb*-TG^Tm4sf5^×Nrf2^mut^ mice were generated by crossbreeding the respective genotype mice. Randomisation was used to allocate experimental units to control and treatment groups to have comparable body weight averages in animal groups.

**Human liver tissue samples:** Liver tissue samples were collected from patients with MASLD at Ewha Womans University Mokdong Hospital following IRB approval (EUMC 2016-07-052). The tissue samples were obtained with informed consents for experimentation with human subjects. The analysis of human tissues has been carried out in accordance with The Code of Ethics of the World Medical Association(Declaration of Helsinki) for experiments involving humans.

**Experimental animals and MASH *in vivo* models:** Eight-weeks-old male C57BL/6 mice (approximately 20 g) were utilized for the experimental protocols. In the HFDCCl_4_ models, mice were placed on a high fat diet (60% kcal fat, TD06414, Teklad, Envigo) and received intraperitoneal injections of carbon tetrachloride (Toronto Research Chemicals, Canada, C176905, 5 mg/kg, twice weekly) over a 12-week period. TSAHC (50 mg/kg in 40% DMSO, administered twice weekly) was injected intraperitoneally to inhibit TM4SF5, following each CCl_4_ treatment. For positive control groups in the MASH model, mice were fed an MCD diet (TD90262, Teklad) for 4 weeks or a MASH diet (A06071302, CDAHFD, Research Diet Inc., New Brunswick, NJ, USA) with L-amino acid rodent formulation containing 60 kcal% fat, 0.1% methionine, and without choline for 12 weeks. To achieve Keap1 gene silencing, mice received intravenous injections of mouse siKeap1 (Assay ID: s78526, Silencer Pre-Designed siRNA, In-Vivo Ready, Ambion, Austin, USA, 0.02 mg/kg formulated in invivofectamine 3.0 reagent, administered twice weekly) for 4 weeks, concurrent with MCD feeding. Control groups maintained a standard chow diet with tap water. Details of the siRNA sequences used are provided in Table 1.

**Isolation and culture of primary hepatocytes:** Prior to preparing the perfusion and digestion buffers, a pre-mixture buffer (10 × Hank's balanced salt solution, 4.2 mM NaHCO_3_, pH 7.0, filtered at 0.2 μM) was made. Throughout all procedures, mice were anesthetized using pads saturated with 30% isoflurane (Terrell, Piramal, PA) dissolved in propylene glycol 200 (8.07483, Sigma-Aldrich). A 24G syringe needle was attached to a peristaltic pump via silicon tubing. The abdominal cavity of the mouse was surgically opened. Perfusion buffer (0.5 mM EGTA in pre-mixture buffer) was administered into the hepatic inferior vena cava at a rate starting from 20 mL/min and reduced to 4 mL/min. Subsequently, the perfusion buffer was replaced with digestion buffer (167 μg/mL Collagenase Type IV in pre-mixture buffer, C5138, Sigma-Aldrich). Livers subjected to digestion were collected and mechanically dispersed through a 100 μm strainer. After centrifuging at 50 ×g for 3 min at 4°C two times, the resulting suspension was collected. Hepatocytes were separated from debris by using a 42% percoll solution (17-0891-02, GE Healthcare). Following removal of the supernatant containing debris, hepatocytes were seeded onto collagen-coated culture dishes and incubated for 24 hrs. The medium used for primary hepatocyte culture was Medium 199, supplemented with 10% FBS, 23 mmol/L HEPES, and 4 ng/mL dexamethasone (265005, Sigma-Aldrich).

**Tissue staining:** Liver tissue paraffin sections from mice were processed with the standard protocol for H/E staining (HEMH-OT and EOYA-10-OT, BIOGNOST). Masson trichrome staining (ab150686, Abcam) was conducted to assess fibrosis following established methods 12. For detection of lipid accumulation, OCT-frozen tissue blocks were stained with Oil red O (O0625, Sigma-Aldrich). Immunohistochemistry (IHC) was performed with antibodies against Keap1 (NBP2-03319, Novus Biologicals), Nrf2 (NBP1-32822, Novus Biologicals), F4/80 (#70076, Cell Signaling Technology), Cd11b (#PA5-79533, Invitrogen), Ccl2 (#MA5-17040, Invitrogen), and α-SMA (A2547, Sigma-Aldrich). IHC stained sections were evaluated using a tissue scanner (Motic Easy Scan Digital Slide Scanner, Motic).

**RNA isolation and real-time (RT) PCR:** Total RNA was purified from cell or mouse liver tissue lysates using Qiazol lysis reagent (79306, QIAGEN, Germanton, MD, USA). cDNA synthesis for qPCR was carried out with a ReverTra Ace™ qPCR RT master mix (Toyobo, Osaka, Japan). Gapdh was consistently used as a reference gene for normalization of sample expression levels. The sequences of the primers employed are detailed in Table 2.

**Luciferase assay:** SNU449 or Huh7 stable cell variant lines were transfected with Keap1 promoter cDNA (HPRM54430-LvPG04, GeneCopoeia™) to assess keap1 promoter activity. Luciferase activity was analyzed using a dual-reporter system based on Gaussia Luciferase (GLuc) and Secreted Alkaline Phosphatase (SEAP) (Secrete-pair™ Dual Luminescence Assay kit, LF032, GeneCopoeia™). Luminescent signals were quantified with a luminometer.

**Protein extraction and western blot:** Cells were washed twice with ice-cold 1 × PBS and subsequently lysed with RIPA buffer (50 mM Tris-HCl, pH 8.0, 150 mM NaCl, 0.1% SDS, 0.5% Sodium deoxycholate, 1% Triton X-100) containing protease inhibitor cocktail (P3100, GenDEPOT) by vortexing on ice for 20 min. Lysates were centrifuged at 12,000 ×*g* for 10 min at 4℃. The supernatant was harvested for protein quantification via BCA assay (Pierce™ BCA Protein Assay Kit, 23225, Thermo Fisher Scientific). For mouse or human liver tissues, samples were lysed in ice-cold RIPA buffer using a temperature-controlled bead homogenizer for 5-10 min followed by centrifugation. Following standard western blot procedures, protein bands were visualized using Image-Quant LAS4000 (GE healthcare Technologies). The primary antibodies applied in this study are detailed in Table 3.

**Subcellular fractionation assay:** Cell extracts were processed to obtain four distinct fractions (cytosolic, membrane, nucleic, cytoskeletal fraction) using the ProteoExtract® Subcellular Proteome Extraction Kit539790 (Millipore) according to the protocol provided by the manufacturer.

**Immunoprecipitation:** Cells (1 × 10^6^) transfected with Flag-, STREP-, or HA-TM4SF5 were lysed using 1% Triton X-100 immunoprecipitation lysis buffer (40 mM HEPES, pH 7.4, 150 mM NaCl, 1 mM EDTA and 0.5% Triton X-100 or 1% Brij58) for 20 min on ice. Part of the supernatant was reserved as input and the remainder was incubated either with primary antibodies for 16 h or with streptavidin-agarose (20353, Thermo Fisher Scientific) for 4 h at 4℃ on a rotator (40 rpm). After antibody incubation, the supernatant was subsequently incubated with agarose bead at 4°C for 2 or 4 hr. Following this, beads were collected by centrifugation at 7500 × *g* for 15 min at 4℃. Beads were washed twice with ice-cold lysis buffer and then with 1 × ice-cold PBS at least three times, after which precipitated proteins were eluted using 2 × SDS-PAGE sample buffer.

**Immunofluorescence:** Cells were seeded onto cover glasses precoated with collagen (1 μg/ml) for 16 hr. One day after seeding, cells were washed with 1 × ice-cold PBS. After fixation with cold 99% methanol, cells were incubated with blocking buffer (5% BSA, 0.3% Triton X-100 in 1 × PBS) at room temperature for 1 hr. The primary antibodies used included TM4SF5-EC2 21 and KEAP1 (sc-365626, Santa Cruz Biotech.). Fluorescence-conjugated secondary antibodies utilized were Alexa Fluor 488 or Alexa Fluor 555 (A21206 or A31570, respectively, Invitrogen). For nuclear staining, DAPI was used. Cover glasses were mounted on glass slides using ProLong™ Gold Antifade (P36930, Invitrogen), and fluorescence imaging was performed using a Nikon Eclipse Ti microscope equipped with a C2 confocal system. Images were analyzed using NIS-Elements software (Nikon, Melville, NY, USA).

**ROS measurement:** For detection of ROS *in vitro*, cells were incubated with H₂-DCFDA (5 μM, D399, Invitrogen™) for 10 to 30 minutes in the dark, collected using trypsin-EDTA, then resuspended in 1× PBS. H2-DCFDA FITC fluorescence was recorded on a flow cytometer (BD FACSLyric, BD Biosciences) at excitation/emission wavelengths of 492-495/517-527 nm. For tissue analysis, frozen liver sections were incubated with dihydroethidium (DHE, 10 μM, D1168, Thermo Fisher Scientific) at room temperature for 15 min in the dark. Stained tissue sections were mounted using ProLong™ Gold Antifade before imaging with a Nikon Eclipse Ti microscope equipped with a C2 confocal system.

**Statistical Analysis:** Statistical analyses were conducted using Prism software version 7.0 (GraphPad, La Jolla, CA, USA). Statistical significance was evaluated by two-way analysis of variance (ANOVA), one-way ANOVA, Mann-Whitney U test, or unpaired Student's *t* test. Differences were considered statistically significant when p < 0.05.

## Results

### TM4SF5-mediated downregulation of protein KEAP1 occurs independently of NRF2 levels in hepatocytes

**Figure 1:**
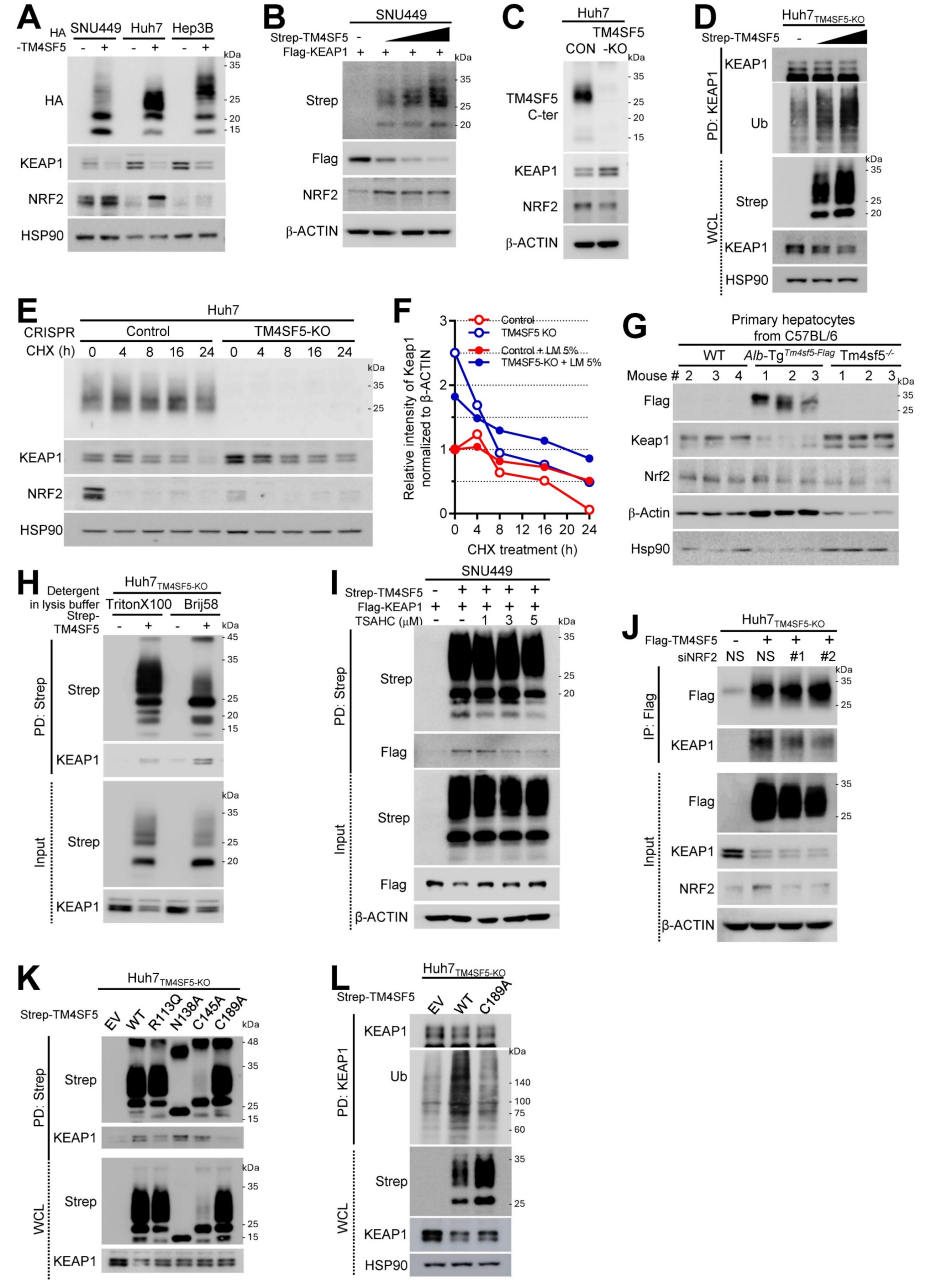
** Hepatocyte TM4SF5-mediated downregulation of protein KEAP1 via their binding is irrelevant to NRF2.** Hepatocytes including TM4SF5-null cell lines [SNU449 lacking endogenously and Huh7_TM4SF5-KO_ via CRISPR/Cas9-mediated KO 47, TM4SF5-expressing cell lines (Hep3B and Huh7_Control_ expressing endogenously), or primary hepatocytes from C57BL/6 male mice (G) were cultured at subconfluent conditions. Cells were transfected either transiently or stably with the relevant cDNAs. Additionally, they were exposed to TSAHC (a TM4SF5-specific chalcone inhibitor) for the indicated durations at varying concentrations **(I)** or transfected with shRNA targeting either a control sequence (shNS) or shNRF2 (target sequences #1 or #2, see Table 1). Whole cell extracts were prepared using lysis buffer containing Triton X-100, Brij58 (H), or Triton X-100 alone (A-G and I-L). Following normalization, proteins were processed for (immuno)precipitation using streptavidin-agarose beads, anti-NRF2 antibody, or anti-KEAP1 antibody, prior to immunoblot analysis for designated molecules. Data shown are representative of three independent experiments. See also Figs. S1 and S2.

To investigate the role of TM4SF5 in modulating ROS during MASLD development, we first assessed the expression levels of NRF2 and KEAP1 in hepatocytes either lacking or expressing TM4SF5. Introducing TM4SF5 into endogenously TM4SF5-deficient SNU449 cells or into TM4SF5-positive Huh7 and Hep3B hepatocytes resulted in decreased KEAP1 and increased NRF2, depending on TM4SF5 expression under basal culture conditions (Fig. 1A). More specifically, exogenous TM4SF5 expression in SNU449 cells led to a reduction in KEAP1 and an elevation in NRF2. Notably, as TM4SF5 levels gradually increased, KEAP1 decreased progressively, while NRF2 upregulation remained unchanged (Fig. 1B). SNU449 stable clones exogenously overexpressing TM4SF5 displayed barely detectable KEAP1 and higher NRF2 compared to control cells (i.e., Cp) (Fig. S1A). Silencing TM4SF5 in Huh7 cells using siTM4SF5 (#2 and #4, see Table 1) elevated KEAP1 levels (Fig. S1B). Genetic knockout of TM4SF5 in Huh7 cells (i.e., Huh7_TM4SF5-KO_) increased KEAP1 but decreased NRF2 expression (Fig. 1C). qRT-PCR analysis of *TM4SF5*, *KEAP1*, and *NRF2* mRNA in stable SNU449 or Huh7 cell lines with empty vector (EV, SNU449_EV_), TM4SF5-HA (SNU449_TM4SF5_), parental control (Huh7_Control_), or TM4SF5-KO (Huh7_TM4SF5-KO_) revealed no significant differences in mRNA expression between TM4SF5-negative and positive variants (Fig. S1C). *KEAP1* promoter activity assays in SNU449 cells co-transfected with varying ratios of TM4SF5 cDNA and *KEAP1* promoter constructs also showed no significant changes (Fig. S1D). Similarly, normal murine AML12 hepatocytes did not exhibit alterations in *Nrf2* or *Keap1* mRNA levels between *Tm4sf5*-negative and *Tm4sf5*-positive lines (Fig. S1E). Huh7_TM4SF5-KO_ cells, with or without TM4SF5 reconstitution, demonstrated TM4SF5-dependent KEAP1 reduction, which was partially reversed by MG132 treatment (Fig. S1F). This TM4SF5-associated decrease in KEAP1 correlated with increased ubiquitination following TM4SF5 expression (Fig. 1D). In contrast, chloroquine (CQ) treatment to inhibit lysosomal degradation did not further affect KEAP1 or NRF2, although KEAP1 levels rose in Huh7_TM4SF5-KO_ cells (Fig. S1G). These findings suggest that TM4SF5-mediated KEAP1 downregulation likely occurs through proteasomal degradation. Following cycloheximide (CHX) treatment to inhibit *de novo* protein synthesis, the elevated KEAP1 in Huh7_TM4SF5-KO_ cells gradually declined. Lower KEAP1 levels observed in Huh7_Control_ cells decreased further with increasing duration of CHX treatment, resulting in a longer half-life of KEAP1 in Huh7_Control_ cells (*t*_50_ = 16 h, open red circles) compared to that in Huh7_TM4SF5-KO_ cells (*t*_50_ = ~ 6 h, open blue circles) (Figs. 1E and 1F). This suggests that TM4SF5 enhances KEAP1 degradation. Inducible KEAP1 suppression using doxycycline (DOX) led to decreased KEAP1 levels, which had been elevated in Huh7_TM4SF5-KO_ cells relative to Huh7_Control_ cells. Conversely, NRF2 levels, which had decreased upon TM4SF5 KO, were restored by further KEAP1 suppression to levels similar to those in Huh7_Control_ cells, indicating that KEAP1 levels were upregulated in Huh7_TM4SF5-KO_ cells (Fig. S1H). Keap1 protein levels demonstrated an inverse relationship with Tm4sf5 expression levels. However, in normal murine AML12 hepatocytes, Nrf2 levels did not significantly change, even though Keap1 was reduced by Tm4sf5 transfection (Figs. S1I). Additionally, SNU449 cells cotransfected with Strep-TM4SF5 and Flag-KEAP1 revealed that TM4SF5 expression resulted in decreased KEAP1 but did not alter NRF2 levels, irrespective of the detergent used in the lysis buffers (Fig. S1J). Remarkably, lysis buffers containing Brij58 or Brij97 detergent were less effective at solubilizing NRF2 compared to KEAP1, in contrast to RIPA (with deoxycholate and NP-40) or CHAPS-based buffers (Fig. S1J). Moreover, primary hepatocytes from WT, hepatocyte-specific Tm4sf5 overexpressing transgenic (TG), or *Tm4sf5*^-/-^ KO C57BL/6 male mice showed that Keap1 levels were lower in TG mice than in either WT or KO mice, while Nrf2 levels did not inversely correlate with Keap1 (Fig. 1G). Additionally, upon lipid mixture (LM, 5%) treatment, KEAP1 protein stability in both Huh7_Control_ and Huh7_TM4SF5-KO_ cells increased comparably to *t*_50_ = ~ 24h, indicating that lipid treatment stabilized KEAP1 to a comparable half-life regardless of TM4SF5 expression (Figs. S1K and 1F, filled circles). Collectively, these findings indicate that TM4SF5 expression can regulate KEAP1 levels independently of NRF2 levels.

### TM4SF5 associates with KEAP1, resulting in the degradation of KEAP1

To further elucidate how TM4SF5 could reduce KEAP1 expression under basal conditions, we first assessed whether TM4SF5 could interact with KEAP1 to facilitate its degradation. We observed that TM4SF5 could indeed bind to KEAP1; however, KEAP1 protein levels were lower in TM4SF5-positive cells compared to TM4SF5-negative cells (Fig. 1H). The binding between TM4SF5 and exogenous Flag-KEAP1 was disrupted by the TM4SF5-specific inhibitor, TSAHC 22, resulting in partial restoration of KEAP1 protein levels upon TM4SF5 expression (Fig. 1I). Fluorescence imaging demonstrated colocalization of TM4SF5 and KEAP1 at endosomal membranes surrounding the perinuclear regions (Fig. S2A).

Although TM4SF5 suppressed KEAP1 expression, the interaction between KEAP1 and NRF2 remained unchanged (Fig. S2B). Notably, reduced KEAP1 levels in TM4SF5-positive Huh7_Conrol_ cells occurred independently of NRF2 overexpression (Fig. S2C, left) or depletion (S2C, right), suggesting that TM4SF5-dependent KEAP1 downregulation is likely unrelated to NRF2 or HO1 expression. In SNU449 cells with NRF2 depletion, varying TM4SF5 expression still resulted in progressive reductions in KEAP1 levels (Fig. S2D). These findings indicate that TM4SF5 expression can alter KEAP1 levels independent of NRF2 expression. In TM4SF5-positive cells, NRF2 suppression modestly decreased the binding affinity between TM4SF5 and KEAP1; however, the interaction persisted, and KEAP1 expression was still reduced following TM4SF5 overexpression regardless of NRF2 status (Fig. 1J). Conversely, formation of the NRF2-KEAP1 complex did not appear to depend on the presence of TM4SF5 (Fig. S2E). Additionally, TM4SF5-KEAP1 binding was abolished by TM4SF5 mutations such as deletions in the C-terminus, N-terminus, or intracellular loop; however, the C-terminus deletion mutant, unlike wildtype (WT) TM4SF5, failed to reduce KEAP1 protein (Fig. S2F). Beyond the deletion mutants, other TM4SF5 point mutants, including R113Q, N138A, C145A, and C189A, also did not cause KEAP1 downregulation, as their KEAP1 levels were similar to control cells lacking TM4SF5 (Fig. S2G, blue highlights). Among these point mutants, the R113Q and C189A variants exhibited reduced binding to KEAP1, which may account for their diminished ability to reduce KEAP1 levels (Fig. 1K). However, the N138A and C145A mutants may not sufficiently lower KEAP1 levels, despite possible binding efficiencies to KEAP1 similar to TM4SF5 WT. These results suggest that the N138A and C145A TM4SF5 mutants' inability to lower KEAP1 is not due to impaired binding, but rather to decreased *N*-glycosylation, reflected as broad bands of 25 ~ 35 kDa on immunoblotting (Fig. 1K). Since KEAP1 in C189A mutant TM4SF5-expressing cells exhibited lower levels of ubiquitination compared to that in TM4SF5 WT cells (Fig. 1L), the reduced binding to KEAP1 may contribute to decreased ubiquitination of KEAP1, in contrast to WT TM4SF5.

### Association of TM4SF5, KEAP1, and CD36 expression in response to extracellular lipid exposure

**Figure 2:**
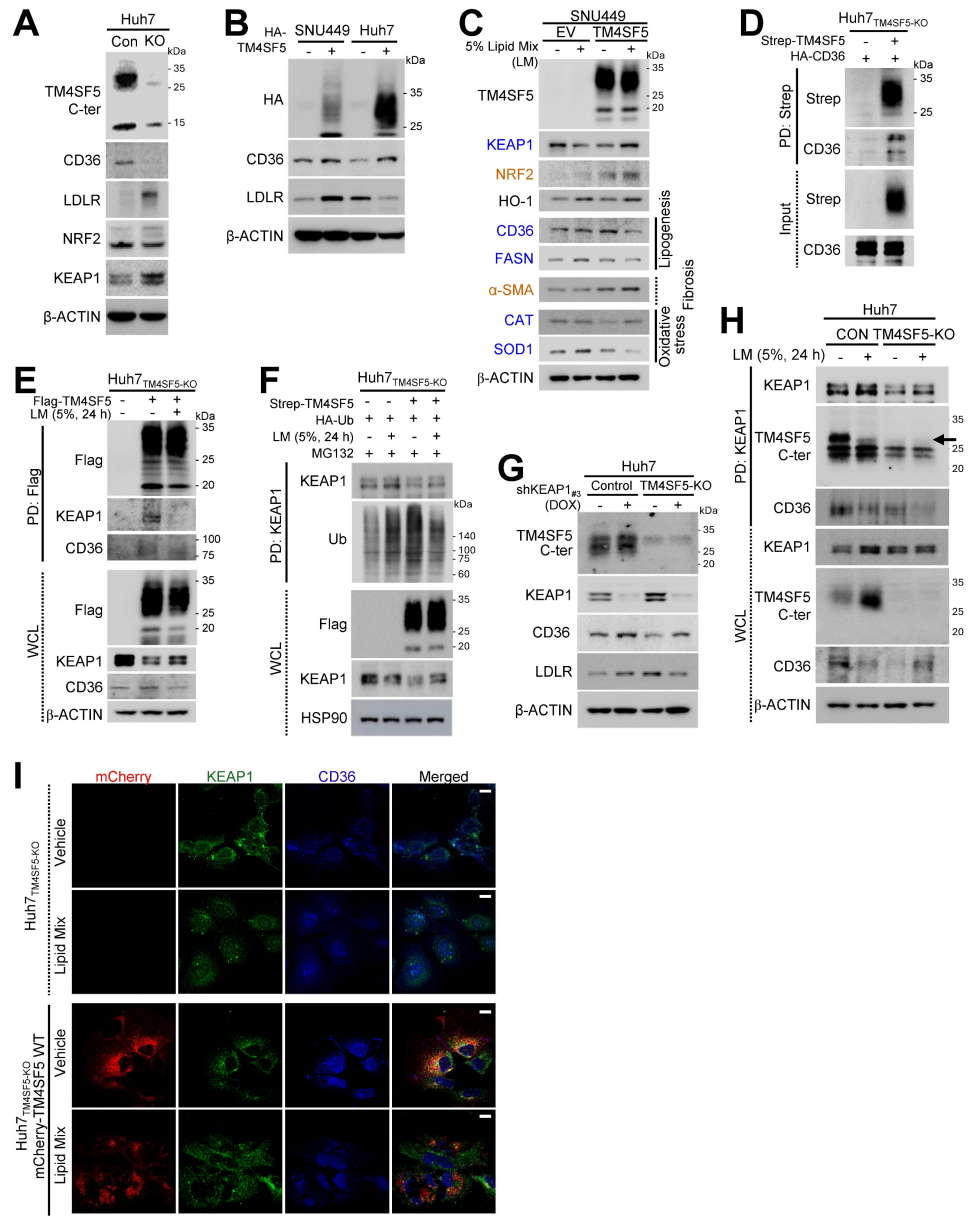
** Correlations among TM4SF5, KEAP1, and CD36 levels following extracellular lipid treatment. (A-H)** Subconfluent Huh7 (A, B, D, E, F, G, H, and I) and SNU449 (B, C) hepatocytes, either left unmodified or subjected to knockout (KO), were stably or transiently transfected with the specified cDNAs. For KEAP1 suppression, cells were treated with doxycycline (DOX) to induce silencing (G). In selected experiments, a lipid mixture (LM, 5%) was administered for 24 h. Following treatment, whole cell lysates (WCL) were prepared using lysis buffer containing Triton X-100. Lysates were quantified and subjected to immunoblotting (A, B, C, and G) or (immuno)precipitation (D, E, F, and H) with streptavidin-agarose beads, anti-Flag, or anti-KEAP1 antibodies, followed by immunoblotting for the indicated proteins. **(I)** Huh7_TM4SF5-KO_ cells complemented with mCherry-TM4SF5 WT were replated onto collagen I-precoated slide glasses and treated with either vehicle or lipid mixture prior to immunostaining and imaging. Scale bar: 100 μm. Data represent three independent experiments. See also Fig. S3.

Since MASLD may involve abnormal lipid accumulation and a hepatic ROS-mediated inflammatory environment via the NRF2-KEAP1 pathway under hyperlipidemic conditions 5, we investigated if TM4SF5-mediated effects are associated with alterations in fatty acid or lipid transporter expression, such as CD36 or LDLR. TM4SF5 knockout (KO) diminished CD36 levels but elevated LDLR and KEAP1 expression (Fig. 2A). Stable overexpression of TM4SF5 in SNU449 or Huh7 cells led to consistent increases in CD36, but LDLR levels displayed cell type-dependent variability, suggesting that TM4SF5-mediated regulation of LDLR may differ between cell lines (Fig. 2B). Notably, palmitic acid (PA) administration to primary hepatocytes from WT C57BL/6 mice raised Keap1 expression (Fig. S3A). Furthermore, exposure to oxidized LDL (oxLDL) or cholesterol in SNU449_EV_ or SNU449_TM4SF5_ cells induced increased KEAP1 with unchanged NRF2 in SNU449_TM4SF5_ cells, whereas the same treatments reduced KEAP1 and elevated NRF2 in SNU449_EV_ cells, in a dose-responsive fashion (Figs. S3B and S3C). In addition, PA-treated AML12 hepatocytes, either unmodified or overexpressing mouse Tm4sf5, displayed Tm4sf5-Keap1 co-localization near perinuclear regions, accompanied by the emergence of vacuole-like cytosolic features indicative of PA-induced cellular stress and lipotoxicity (Fig. S3D). Moreover, in SNU449 cells, TM4SF5 expression decreased KEAP1 protein, whereas additional lipid mixture treatment caused decreased KEAP1 in control SNU449_EV_ or increased KEAP1 in TM4SF5-expressing SNU449_TM4SF5_ cells, respectively (Fig. 2C). Changes in NRF2 or heme oxygenase-1 (HO-1) under these conditions were not associated with KEAP1 alterations; however, CD36 and fatty acid synthase (FASN) showed expression changes inversely related to KEAP1, depending on TM4SF5 status and lipid mixture exposure (Fig. 2C). Unlike catalase (CAT), superoxide dismutase 1 (SOD1) expression was consistent with the pattern of CD36 and FASN, whereas α-smooth muscle actin (SMA), a fibrotic marker, paralleled NRF2 expression (Fig. 2C). Treatment with palmitic acid (PA) or a lipid mixture (LM) at varying concentrations resulted in lipid-induced reductions or elevations of KEAP1 in SNU449_EV_ or SNU449_TM4SF5_ cells, respectively; These KEAP1 alterations corresponded with changes in SIRT1 and DGAT1, whereas NRF2 modulation showed associations with HO-1 or NQO1 levels (Figs. S3E and S3F).Given that SIRT1 and DGAT1 are implicated in inflammatory processes during MASH development 12 or in lipogenesis 23 and that SOD1 deficiency is associated with nonalcoholic fatty liver [NAFL, 24, these TM4SF5-mediated influences on such molecules upon lipid exposure may result in abnormal lipid accumulation through enhanced lipid uptake and intracellular synthesis, ultimately contributing to MASLD. In addition, TM4SF5-mediated modulation of KEAP1 also appeared to affect these features of MASLD. We further observed that TM4SF5 binds to HA-CD36, indicating that TM4SF5-associated CD36 in hepatocytes may be inversely related to the TM4SF5-driven reduction in KEAP1 expression (Fig. 2D). Lipid mixture (LM) treatment led to dissociation of KEAP1 binding from TM4SF5, with a slight restoration in KEAP1 levels, while the interaction of CD36 with TM4SF5 was modestly decreased in parallel with reduced CD36 expression in TM4SF5-positive cells after lipid exposure (Fig. 2E). Treatment of TM4SF5-null cells with lipids resulted in KEAP1 levels being somewhat higher (or restored) compared to the decreased KEAP1 levels observed with TM4SF5 expression alone, and this alteration may be attributed to KEAP1 ubiquitination (Fig. 2F). Furthermore, other fatty acid transporters, namely FABP1, FABP3, and LDLR, were analyzed for their ability to bind TM4SF5; FABP1/3 were found to interact with TM4SF5, whereas LDLR did not (Figs. S3G, S3H, and S3I).

### TM4SF5 interacts with CD36 following extracellular lipid exposure, leading to KEAP1 stabilization

We further investigated the role of KEAP1 in regulating TM4SF5-dependent CD36 expression. Inducible knockdown of KEAP1 in Huh7 cell variants resulted in elevated CD36 expression in both Huh7_Control_ and Huh7_TM4SF5-KO_ cells; however, CD36 levels remained higher in Huh7_Control_ cells than in Huh7_TM4SF5-KO_ cells. Additionally, in Huh7_Control_ cells, CD36 expression was positively correlated with LDLR levels, whereas this relationship was inverse in Huh7_TM4SF5-KO_ cells (Fig. 2G). In contrast, overexpression of Flag-KEAP1 in Huh7_Control_ cells led to decreased CD36 and LDLR expression, while TM4SF5 knockout resulted in decreased CD36, yet increased LDLR levels (Fig. S3J). Given that LDLR did not interact with TM4SF5 or KEAP1 (Figs. S3H and S3I) and showed no correlation with TM4SF5 (Figs. 2B and S3J), we directed our attention towards CD36 and excluded LDLR from further consideration. Notably, CD36 was found to associate with KEAP1 (Figs. S3K and S3L). KEAP1 immunoprecipitates from Huh7 cell variants, either untreated or subjected to LM exposure, indicated efficient formation of a ternary complex (TM4SF5, KEAP1, and CD36); in Huh7_Control_ cells, lipid treatment reduced the association of both CD36 and TM4SF5 with KEAP1. Meanwhile, in Huh7_TM4SF5-KO_ cells, KEAP1 still interacted with CD36, though to a lesser extent than in untreated Huh7_Control_ cells, with binding further decreased after lipid treatment (Fig. 2H). Similarly, colocalization of KEAP1, CD36, and TM4SF5 near perinuclear regions was diminished in Huh7_TM4SF5-KO_ cells reconstituted with mCherry-TM4SF5 WT following lipid mixture exposure (Fig. 2I). Collectively, these findings indicate that TM4SF5 influences KEAP1 and CD36 expression differently in basal versus lipid-exposed conditions, possibly contributing to dysregulated lipid accumulation via dynamic protein complexes comprising TM4SF5, KEAP1, and/or CD36 in TM4SF5-positive hepatocytes.

### KEAP1 stabilization mediated by TM4SF5 in lipid-exposed hepatocytes promotes oxidative stress

**Figure 3:**
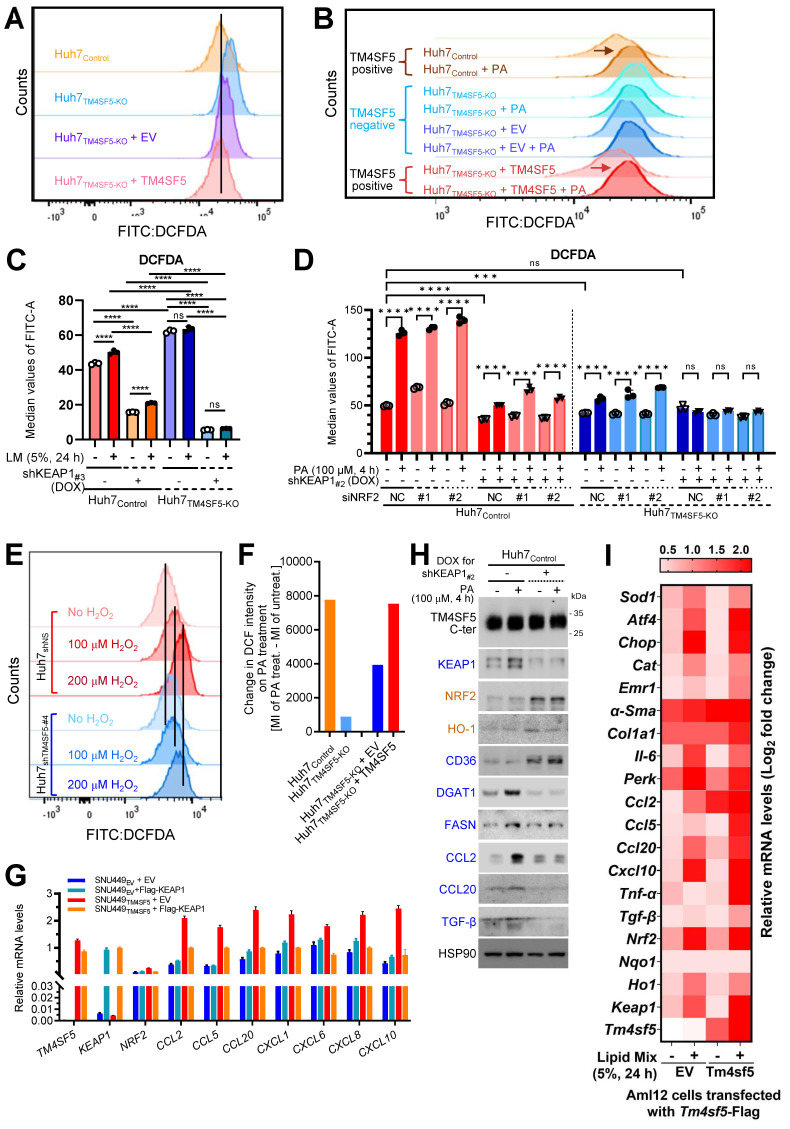
** TM4SF5-mediated stabilization of KEAP1 in lipid-treated hepatocytes promotes oxidative stress and hepatic inflammation. (A-F)** Subconfluent Huh7_Control_ or Huh7_TM4SF5-KO_ hepatocyte variants, either stably or transiently transfected with the indicated cDNAs, were analyzed for ROS production using flow cytometry following DCFDA staining. Cells received vehicle treatment (-), PA alone (B, D, and F), lipid mixture (LM, C), or DOX to induce Keap1 knockdown (shKEAP1_#2 or #3_, see Table 1, C and D). In some experiments, Huh7 cell variants were transfected with siNS (non-specific sequences as a control) or siNRF2 (targeting sequence #1 or #2, see Table 1) for 24 h. Cells were then treated with DOX for shKEAP1_#2_ induction for 24 h, followed by PA for 4 h prior to ROS analysis by flow cytometry (D). Cells were also exposed to H_2_O_2_ at the indicated concentrations and durations before assessment of ROS levels (E). *, *P* < 0.05; **, *P* < 0.01; ***, *P* < 0.001; ***, *P* < 0.0001; ns = non-significant, unpaired Student's *t* test or two-way ANOVA. Data are expressed as mean ± SEM. **(G)** Subconfluent SNU449 hepatocytes stably expressing empty vector (SNU449_EV_) or TM4SF5 (SNU449_TM4SF5_) were harvested to perform qRT-PCR for the indicated molecules. **(H)** Subconfluent Huh7_Control_ cells were treated in the absence (-) or presence (+) of DOX to induce KEAP1 knockdown (shKEAP1_#2_) for 24 h and then exposed to PA (100 μM) for 4 h before being collected for immunoblot analysis of the indicated molecules. **(I)** Murine AML12 cells stably transfected with EV or TM4SF5 were treated with 5% LM for 24 h, harvested, and subjected to qRT-PCR for the indicated molecules. Data shown are representative of three independent experiments. See also Fig. S4.

We next examined how TM4SF5-mediated alterations in KEAP1 expression may relate to intracellular ROS levels. FACS analysis demonstrated that baseline ROS concentrations were lower in TM4SF5-positive Huh7 cells compared to their TM4SF5-negative counterparts (Fig. 3A). Notably, TM4SF5-positive Huh7 cells exhibited substantial increases in ROS following PA treatment, in contrast to TM4SF5-negative cells, which displayed either minimal changes or slight reductions in ROS (Fig. 3B). Huh7_Control_ cells showed increased ROS levels in response to lipid mixture treatment, whereas Huh7_TM4SF5-KO_ cells exhibited negligible changes, although their ROS levels remained higher than those observed in Huh7_Control_ cells. Suppressing KEAP1 led to a marked reduction in ROS levels, with a more pronounced decrease in Huh7_TM4SF5-KO_ cells (Fig. 3C). Interestingly, ROS levels in Huh7_TM4SF5-KO_ cells remained unchanged upon lipid treatment (Fig. 3C). When compared with Huh7_TM4SF5-KO_ cells, Huh7_Control_ cells demonstrated an elevation in ROS levels upon PA treatment; this elevation was notably diminished with KEAP1 suppression but was not affected by NRF2 suppression (Fig. 3D). In Huh7_TM4SF5-KO_ cells with KEAP1 suppression, PA treatment failed to affect ROS levels, regardless of NRF2 suppression (Fig. 3D). Extracellular H_2_O_2_ treatment of Huh7 cells, with or without TM4SF5 suppression, produced greater ROS accumulation in non-suppressed Huh7 cells than in cells where TM4SF5 was suppressed, and this effect was more pronounced in TM4SF5-expressing Huh7 cells (Fig. 3E). Moreover, PA treatment led to greater alterations in ROS levels in TM4SF5-positive cells as opposed to TM4SF5-negative counterparts (Fig. 3F). Collectively, these findings indicate that TM4SF5-mediated modulation of KEAP1 levels results in more prominent changes in ROS, with these effects being less affected by or independent of NRF2 expression.

### TM4SF5-mediated KEAP1 stabilization in lipid-treated hepatocytes promotes inflammation

We further examined whether TM4SF5-mediated changes in KEAP1 levels were associated with the induction of cytokines or chemokines relevant to hepatic inflammation. Notably, SNU449_TM4SF5_ and Huh7_Control_ cells demonstrated elevated mRNA expression of various cytokines/chemokines, including *CCL2*, *CCL5*, *CCL13*, *CCL20*, *CXCL2*, *CXCL3*, *CXCL12*, among others, compared to SNU449_EV_ and Huh7_TM4SF5-KO_ cells, respectively, even though *KEAP1* and *NRF2* mRNA expression patterns differed (Figs. S4A and S4B). SNU449 and Huh7 cell variants transfected with EV or Flag-KEAP1 revealed that cytokines/chemokines upregulated by TM4SF5, including *CCL2*, *CCL5*, *CCL20*, *CXCL1*, *CXCL6*, *CXCL8*, and *CXCL10*, were substantially decreased by additional Flag-KEAP1 expression, in contrast to SNU449_EV_ or Huh7_TM4SF5-KO_ cells, where the changes were minimal or ineffective, whereas *NRF2* levels remained unchanged under these experimental conditions (Figs. 3G and S4C). Huh7_Control_ cells without KEAP1 suppression displayed a more pronounced induction of cytokines/chemokines following PA treatment compared to cells in which KEAP1 was suppressed (Fig. S4D). The expression profiles of these cytokines/chemokines were consistent with those of *TM4SF5* and *KEAP1*, but not *NRF2* (Fig. S4D). CCL2, CCL5, CCL20, and CXCL10 have been implicated in generating a pro-inflammatory milieu during TM4SF5-mediated MASLD 12. When either vehicle or PA was applied to Huh7_Control_ cells with or without inducible KEAP1 suppression, KEAP1 protein expression positively correlated with DGAT1, FASN, CCL2, CCL20, and TGFβ, but negatively with CD36, whereas NRF2 correlated with HO-1 (Figs. 2C and 3H). In normal AML12 murine hepatocytes with stable expression of EV or Tm4sf5, lipid mixture treatment resulted in increased mRNA expression of genes associated with ROS generation, inflammation, fibrosis, and stress, including *Keap1*, *Ccl2*, *Ccl5*, *Ccl20*, and additional targets. Notably, these increases were more pronounced in AML12_Tm4sf5_ cells than in AML12_EV_ cells (Fig. 3I). Additionally, PA treatment elevated *Nrf2* mRNA levels in both AML12_EV_ and AML12_Tm4sf5_ cells to similar extents (Fig. 3I). These findings indicate that TM4SF5-mediated upregulation of KEAP1, in response to extracellular lipid treatment, may contribute to elevated cytokine and chemokine production linked to hepatic inflammation.

### *In vivo* MASH-associated fibrosis models demonstrate TM4SF5-dependent stabilization of KEAP1

**Figure 4:**
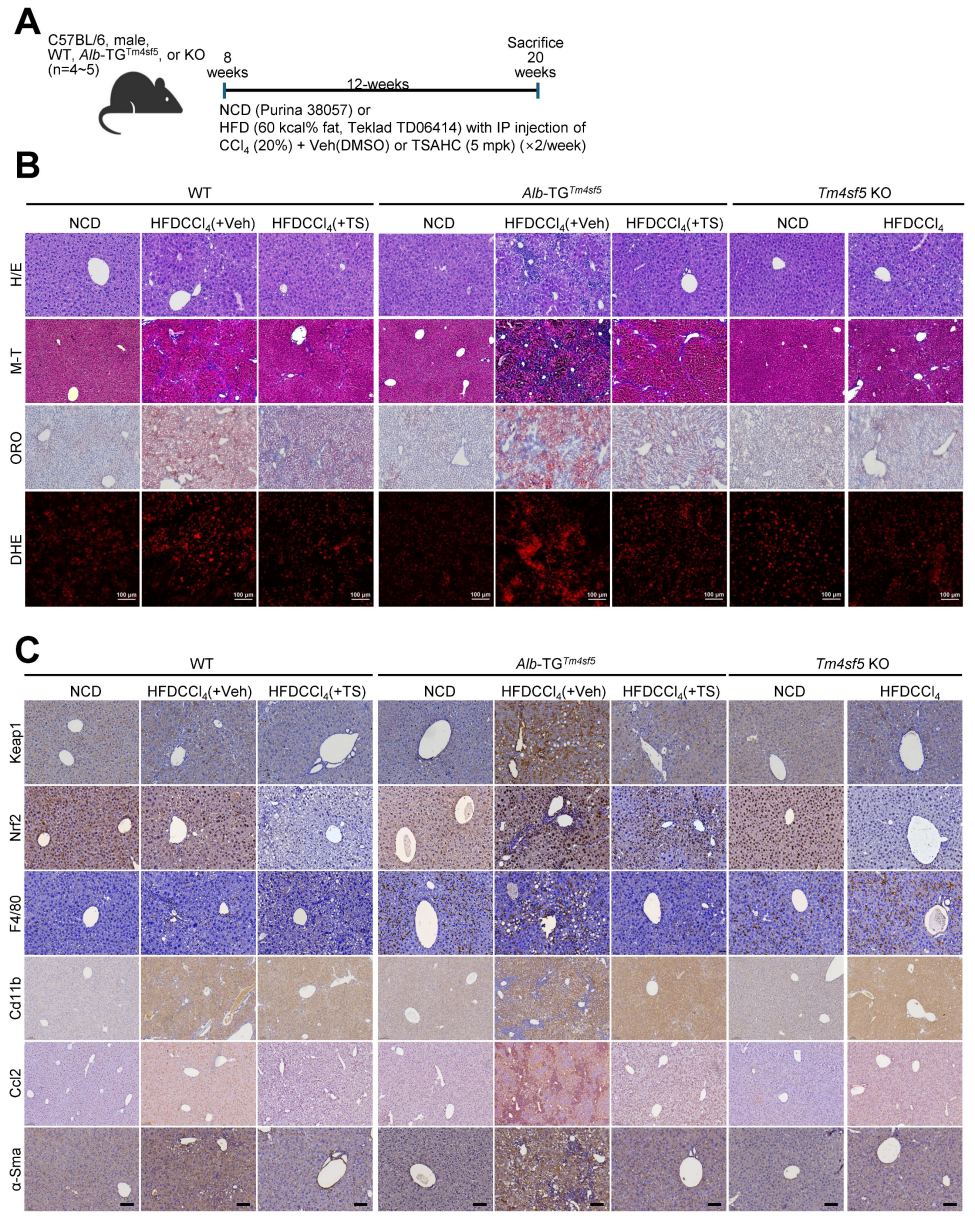

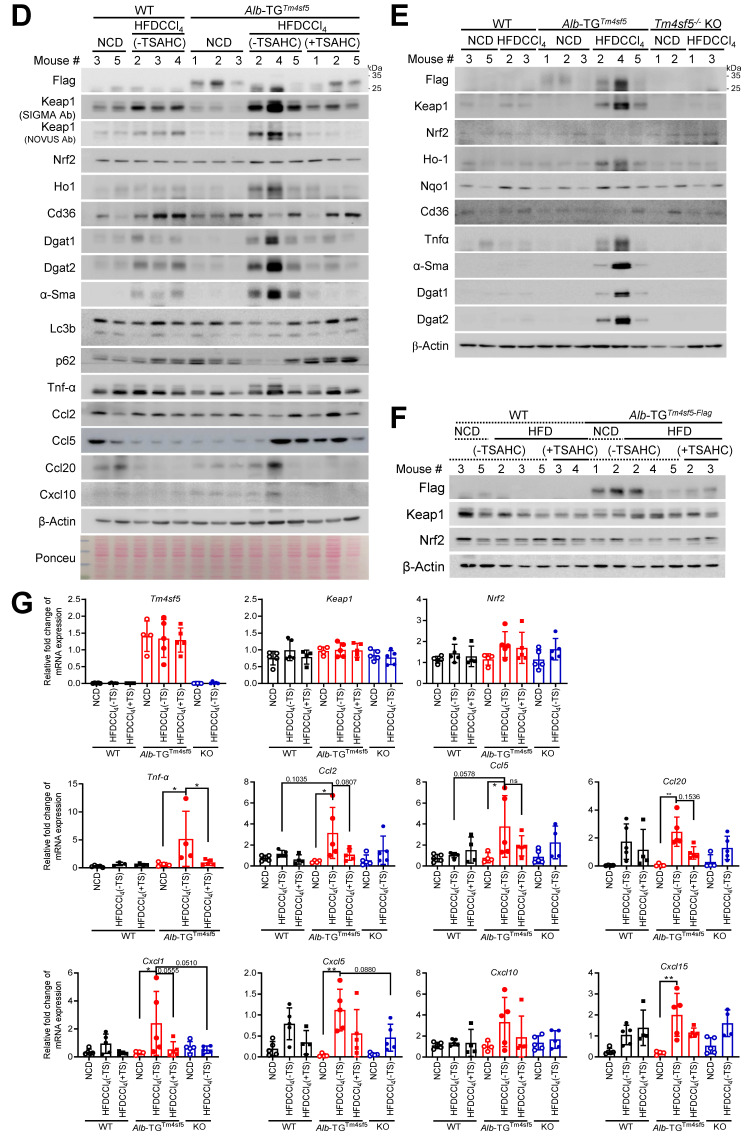
***In vivo* MASH-associated fibrosis model involves TM4SF5-dependent KEAP1 stabilization. (A-E)** WT, *Alb*-TG^Tm4sf5-Flag^, and *Tm4sf5*^-/-^ KO C57BL/6 male mice (n=4~5, 8-week-old) were fed NCD or HFD without or with CCl_4_ IP injection (HFDCCl_4_) in the presence of vehicle DMSO or TSAHC treatment (IP injection, twice per week) for an additional 12 weeks until 20 weeks of age (A), after which they were euthanized for liver analysis using H&E, Masson's trichrome (M-T), Oil Red O (ORO), dihydroethidium (DHE) staining (B), immunohistochemistry (C), or immunoblotting (D and E). Scale bar: 100 μm.** (F)** Livers from animals fed only NCD or HFD for 12 weeks were processed to prepare whole tissue extracts for subsequent immunoblotting targeting the indicated molecules. **(G)** Liver tissues from animals treated as in (A) were analyzed by qRT-PCR for the target molecules. *, *P* < 0.05; **, *P* < 0.01; ***, *P* < 0.001; ***, *P* < 0.0001; ns, non-significant, unpaired Student's *t* test or two-way ANOVA. Data are presented as mean ± SEM. Data shown are representative of three independent experiments. See also Fig. S4.

We subsequently evaluated whether hepatocyte TM4SF5 expression mediates KEAP1 level-dependent modulation of ROS during MASH-associated fibrosis in murine livers. We exposed 8-week-old male C57BL/6 mice (WT, *Alb*-TG^Tm4sf5-Flag^, or KO; n = 4~5) maintained on either normal chow diet (NCD) or high-fat diet (60 kcal% fat, HFD) to CCl_4_ injection in the presence of either vehicle DMSO or TSAHC (administered twice weekly) for a duration of 12 weeks (Fig. 4A). In contrast to NCD-fed mice, WT and *Alb*-TG^Tm4sf5^ animals on HFDCCl_4_ with vehicle exhibited pronounced liver injury, collagen I deposition, excessive lipid accumulation, and elevated ROS, all indicative of MASH-associated fibrosis. These pathological features were abolished following TSAHC intervention, whereas KO mice did not manifest these characteristics (Fig. 4B). Immunostaining for Keap1, Nrf2, F4/80, Cd11b, Ccl2, and α-smooth muscle actin (Sma) further corroborated the fibrotic changes (Fig. 4C). Markedly increased Keap1 protein levels in *Alb*-TG^Tm4sf5^ mice under HFDCCl_4_ without TSAHC treatment correlated with regulators of lipogenesis and inflammation, but not with Nrf2 expression. These associations were also observed in WT mice but not KO mice (Figs. 4D and 4E). The elevation of Keap1 in TM4SF5-expressing hepatocytes suggests a possible connection to dysregulated lipid metabolism and sustained hepatic inflammation. Moreover, acute LPS exposure in isolated primary hepatocytes did not significantly impact ROS levels, independent of Tm4sf5 status (Fig. S4E). HFD alone is insufficient to evoke TM4SF5-mediated Keap1 changes leading to MASH-associated fibrosis (Fig. 4F), supporting the notion that HFD cannot cause MASH 25. Notably, though increased mRNA levels of inflammatory cytokines and chemokines were observed in *Alb*-TG^Tm4sf5^ mice subjected to HFDCCl_4_, subsequent TSAHC administration only moderately or insignificantly reduced these transcripts (Fig. 4G), and these discrepancies correlated predominantly with marked differences in Keap1 protein rather than *Keap1* mRNA levels (Figs. 4D, 4E, and 4G).

### Murine and human MASH models implicate Tm4sf5, Keap1, and CD36, likely independent of NRF2's DNA-binding function

**Figure 5:**
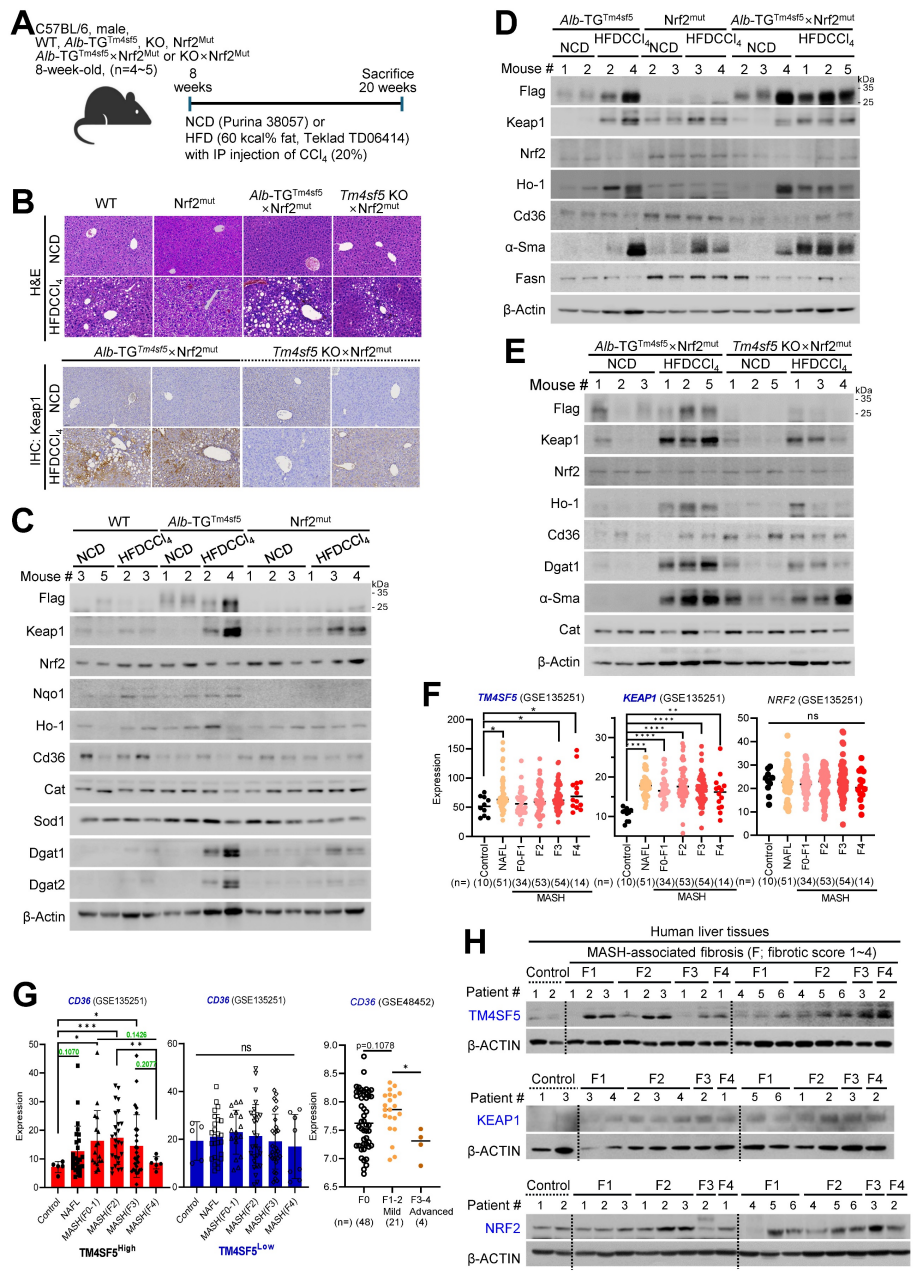
** Both murine and human MASH models demonstrate involvement of Tm4sf5, Keap1, and CD36, likely independent of the DNA-binding ability of NRF2. (A-E)** WT, Nrf2^mut^, *Alb*-TG^Tm4sf5^×Nrf2^mut^, *Tm4sf5*^-/-^ KO×Nrf2^mut^ male C57BL/6 mice (n = 4~5, 8-week-old) were fed either NCD or HFD (60% kcal fat) alongside intraperitoneal CCl_4_ injection (5 mg/kg, twice per week) for 12 weeks, after which tissues were examined using H&E staining or immunohistochemistry for Keap1 (B), or subjected to immunoblotting for selected proteins (C-E). **(F and G)** Analyses of GSE135251 dataset from either healthy controls or MASLD patients with varying NAS (from 0 to 4) or GSE48452 dataset was conducted to compare gene expression levels among normal, NAFL, and MASH patient samples stratified by NAS. The TM4SF5^High^ or TM4SF5^Low^ classification was determined based on the top 50% or bottom 50% expression levels of *TM4SF5* mRNA in the cohort. **(H)** Human liver biopsy samples from control individuals and MASLD patients with different NAS (from 0 to 4) were used to obtain whole tissue extracts for immunoblotting of designated proteins. Because biopsy samples were very small (~ 7 mm circular pillar with 1 mm diameter), sufficient protein extracts for all targets were not always available, resulting in variable patient numbers analyzed in each immunoblot. *, *P* < 0.05; **, *P* < 0.01; ***, *P* < 0.001; ***, *P* < 0.0001; ns, non-significant, one-way ANOVA. Data are reported as mean ± SEM. Findings presented represent three independent experimental replicates. See also Fig. S5.

Next, we examined the involvement of Nrf2 in TM4SF5-mediated effects by utilizing animals harboring an Nrf2 mutant incapable of binding DNA due to a deletion in the CNC bZIP region (amino residues 452 to 560) 20. Nrf2^Mut^ C57BL/6 male mice under the HFDCCl_4_ condition (Fig. 5A) developed hepatocyte damage, but, in contrast to WT mice displaying MASH features, did not exhibit fat droplet deposition (Fig. 5B). However, mice generated from crossing *Alb*-TG^Tm4sf5^ with Nrf2^Mut^ (*Alb*-TG^Tm4sf5^×Nrf2^Mut^) exhibited severe hepatocyte damage, pronounced fat droplet accumulation, and local immune cell infiltration, whereas those crossed between *Tm4sf5*^-/-^ KO and Nrf2^Mut^ (*Tm4sf5*^-/-^ KO×Nrf2^Mut^) presented substantially reduced severity of these features; Keap1 levels were also markedly elevated in *Alb*-TG^Tm4sf5^×Nrf2^Mut^ mice compared to *Tm4sf5*^-/-^ KO×Nrf2^Mut^ mice under HFDCCl_4_ conditions (Fig. 5B). Western blot analysis of liver extracts further revealed that HFDCCl_4_ exposure led to greater increases in Keap1 in *Alb*-TG^Tm4sf5^ mice than in Nrf2*^Mut^* mice, with concurrent elevation of DGAT1/2 levels (Fig. 5C). Furthermore, Keap1 levels in *Alb*-TG^Tm4sf5^×Nrf2^Mut^ mice exposed to HFDCCl_4_ exceeded those observed in *Tm4sf5*^-/-^ KO×Nrf2^Mut^ mice, accompanied by increased α-Sma expression, which is indicative of fibrotic progression (Figs. 5D and 5E). A similar weak association with MASH features in Nrf2^Mut^ mice was also detected in those fed a MASH diet (CDAHFD) for 12 weeks, although *Alb*-TG^Tm4sf5^×Nrf2^Mut^ mice developed extensive fat accumulation and inflammation at levels comparable to *Alb*-TG^Tm4sf5^ mice (Fig. S5A).

In an MASLD model induced by MCD diet, hepatic Cd36 levels are inversely correlated with Keap1 levels 26, as also observed in *Alb*-TG^Tm4sf5^mice or *Alb*-TG^Tm4sf5^×Nrf2^Mut^ mice under HFDCCl_4_ conditions (Figs. 5C-5E). Analysis of public datasets revealed pronounced positive correlations between hepatic *TM4SF5* and *KEAP1* mRNA levels in human MASH patients (GSE135251), as compared with the control group; by contrast, *NRF2* levels did not show a significant correlation with, nor substantial changes as, steatohepatitic features or fibrosis score escalated (Fig. 5F). Among MASLD patients, individuals in the *TM4SF5*^High^ group (top 50%) exhibited more pronounced alterations in *CD36* expression than the *TM4SF5*^Low^ group (bottom 50%, Fig. 5G). In addition, the TM4SF5^High^ group demonstrated a significant rise in *CD36* mRNA levels as the fibrosis score progressed up to F2, with a subsequent decline at F3~4, whereas the TM4SF5^Low^ group did not present comparable changes in *CD36* compared to controls (Fig. 5H). Notably, the mean *CD36* mRNA levels among MASH patients (across F0 to F4) exhibited a biphasic pattern in GSE135251 and GSE48452 dataset as well (Fig. 5G). Furthermore, TM4SF5 protein levels demonstrated a correlation with KEAP1 but not with NRF2 protein, remaining proportional to fibrotic scores in liver tissue extracts from individuals with MASH-associated fibrosis, in contrast to control samples (Fig. 5H).

We further examined whether exposure to a methionine-choline deficient (MCD) diet alone in a MASH model could influence Keap1 via Tm4sf5-mediated mechanisms. C57BL/6 male mice (8-week-old, n=5) were fed either NCD or MCD diets for 4 weeks before evaluation of their livers (Fig. S5B). Larger fat droplets, increased immune cell infiltration, and elevated Keap1 immunostaining were evident following MCD diet administration in WT and *Alb*-TG^Tm4sf5-Flag^ mice, but not in KO mice (Fig. S5C). *Alb*-TG^Tm4sf5^ mice given the MCD diet exhibited heightened Keap1 expression and upregulation of molecules associated with lipid synthesis and inflammation (Fig. S5D). Although hepatocyte TM4SF5 appears to upregulate KEAP1 during sustained ROS production and inflammation leading to MASH-associated fibrosis, NRF2 may be less central in modulating ROS and inflammatory processes.

### Keap1 suppression abolishes TM4SF5-mediated MASH-associated fibrosis induced by an MCD diet

**Figure 6:**
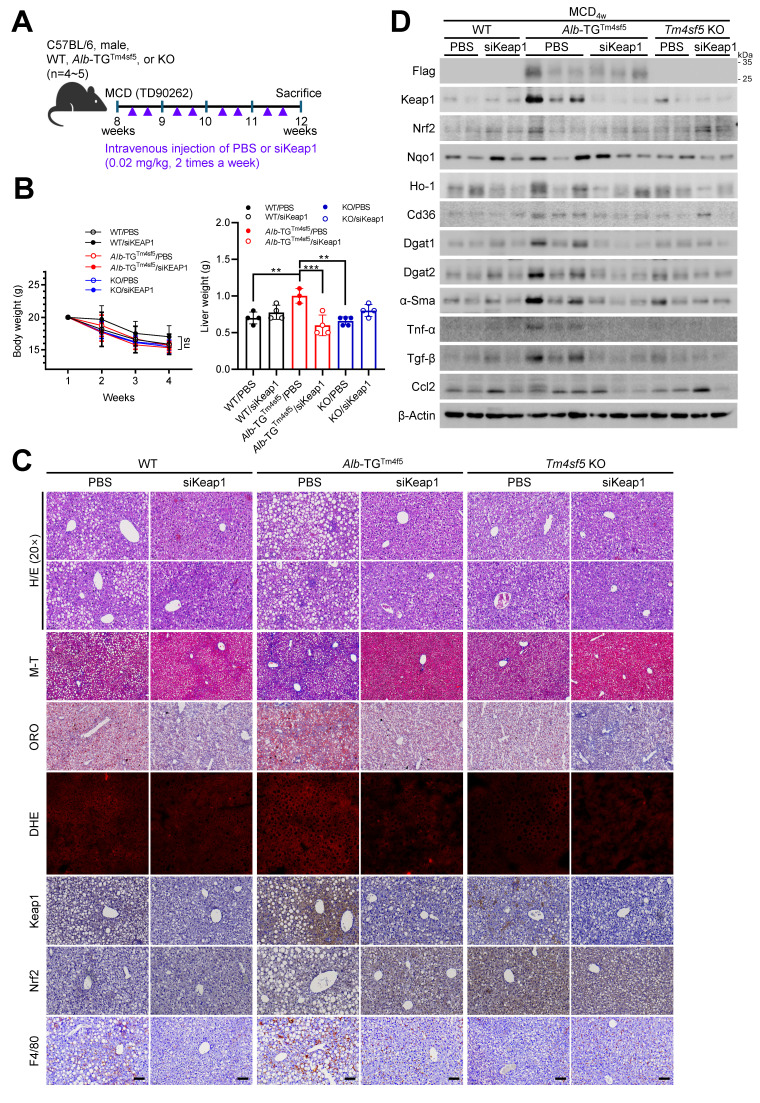
** Keap1 downregulation reverses TM4SF5-driven, MASH-associated fibrosis upon MCD diet. (A-D)** WT, *Alb*-TG^Tm4sf5-Flag^, and *Tm4sf5*^-/-^ KO C57BL/6 male mice (n=4~5, 8-week-old) were maintained on an MCD diet for 3.5 weeks (A) and received intravenous injections of either PBS or siKeap1 (0.02 mg/kg, twice a week) prior to sacrifice for subsequent hepatic analyses, including body and liver weight measurements (B), and evaluation through H&E, Masson's trichrome (M-T), Oil Red O (ORO), or dihydroethidium (DHE) staining, immunohistochemistry (C), or immunoblotting (D) for specified proteins. Scale bar: 100 μm. *, *P* < 0.05; **, *P* < 0.01; ***, *P* < 0.001; ***, *P* < 0.0001; ns, non-significant, one-way ANOVA. All data are expressed as mean ± SEM.

We subsequently investigated whether Keap1 suppression could prevent the TM4SF5-mediated development of MASLD characteristics induced by an MCD diet. C57BL/6 male mice (8-week-old, n = 4~5) received an MCD diet for 4 weeks, accompanied by intravenous injections of PBS or siKeap1 (2 times/week) (Fig. 6A). As anticipated, administration of the MCD diet led to body weight loss; notably, *Alb*-TG^Tm4sf5^ mice exhibited significantly higher liver weights compared with WT or KO mice, a difference that was mitigated by concurrent Keap1 suppression (Fig. 6B). Notably, H&E and immunohistochemical analyses demonstrated that *Alb*-TG^Tm4sf5^ mice developed more pronounced hepatic fat accumulation, collagen I deposition, ROS production, immune cell infiltration, and Keap1 expression relative to WT mice; however, all these alterations were reversed following Keap1 suppression, while KO mice did not display these phenotypes (Fig. 6C). Immunoblotting of liver samples showed elevated Keap1 expression and increased levels of proteins associated with lipid uptake, synthesis, inflammation, and fibrosis in *Alb*-TG^Tm4sf5^ mice fed the MCD diet, yet these increases were negated by Keap1 suppression. Conversely, such molecular changes were not detected in KO mice (Fig. 6D). Collectively, these findings suggest that the TM4SF5-dependent upregulation of Keap1 may contribute to the regulation of chronic dysregulated lipid uptake/metabolism, ROS generation, and inflammation during MASLD progression.

## Discussion

**Figure 7:**
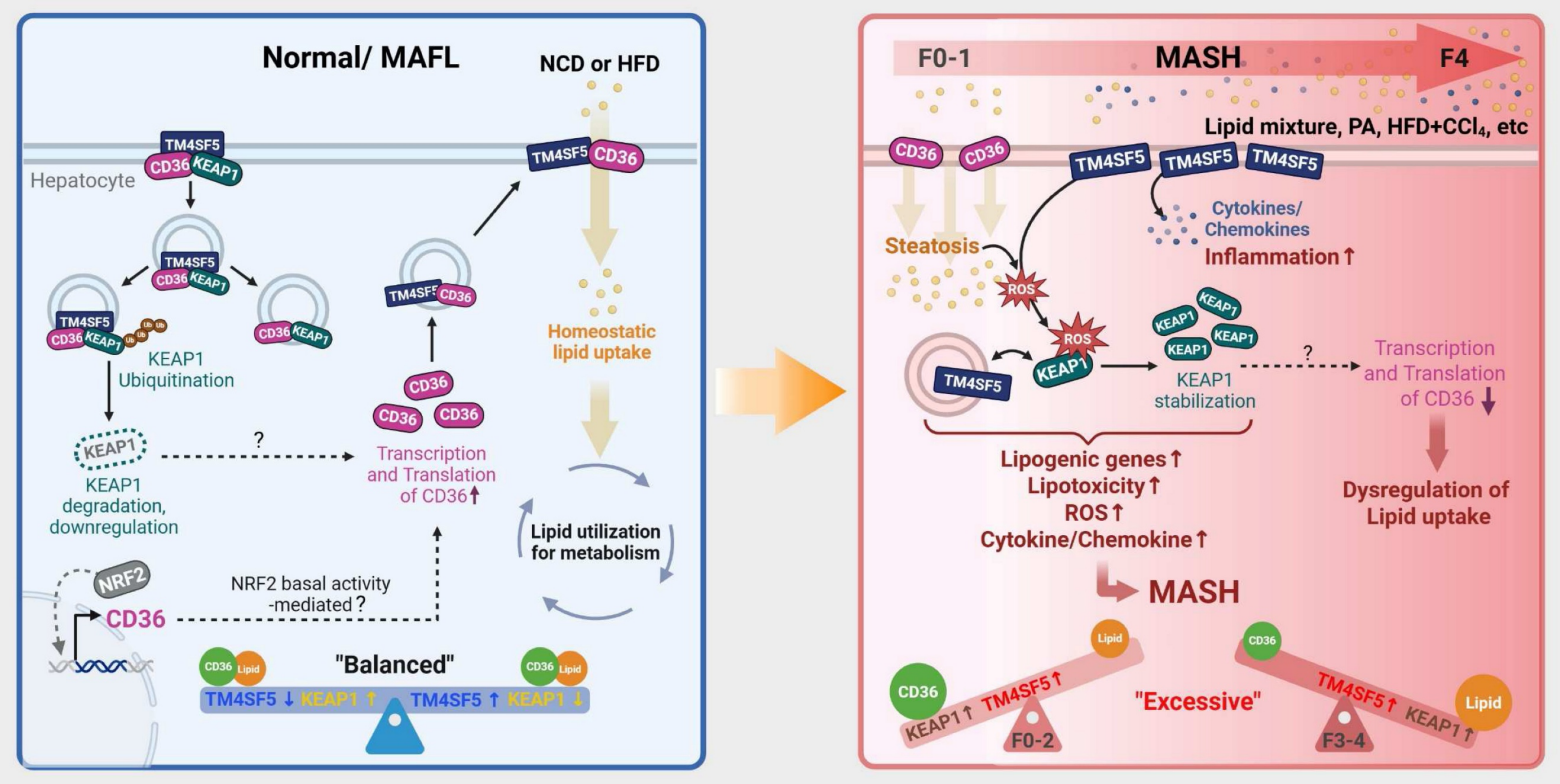
** Schematic model illustrating the modulation of KEAP1 by TM4SF5 under basal and pathological conditions.** (Left) In healthy livers, TM4SF5 expression is very low and can be further upregulated by a hepatic inflammatory environment, presumably during early homeostatic conditions with minimal features of fatty liver. In this context, KEAP1 maintains regulation of NRF2 for antioxidant gene induction. In livers exhibiting balanced lipid catabolism and anabolism, or in the presence of extracellular lipids that do not induce MASH [e.g., HFD 25, low levels of tetraspanin TM4SF5 associate with KEAP1 and CD36, promoting KEAP1 ubiquitination and degradation and consequently increasing CD36 levels, thereby supporting homeostatic lipid metabolism. (Right) In situations of greater extracellular lipid overload, which can trigger MASH, TM4SF5 enhances the production of inflammatory cytokines and chemokines and increases ROS accumulation. Concurrently, TM4SF5 expression is elevated due to inflammation and further stabilizes KEAP1, facilitating ROS sensing and the induction of lipogenic genes. This leads to dysregulated lipid uptake, likely due to decreased CD36 levels, and an increase in NAS (from F1 to F4). Accordingly, dysregulated lipid metabolism and the pro-inflammatory environment, including ROS accumulation in TM4SF5-positive hepatocytes or livers, can drive MASH through TM4SF5-mediated KEAP1 stabilization, presumably independent of NRF2 levels or transcriptional function.

This study demonstrated that TM4SF5 in hepatocytes may reduce KEAP1 expression independently of NRF2 levels under basal conditions, but can increase KEAP1 expression in lipid-induced pathological states, likely by regulating KEAP1 ubiquitination or stability through TM4SF5-KEAP1 complex assembly. The interaction between TM4SF5 and KEAP1 further engaged CD36 for enhanced association in *in vitro* models, WT or *Alb*-TG^mTm4sf5-Flag^ mice receiving HFDCCl_4_, and human MASLD specimens, concomitant with the formation of a tripartite complex in basal states. These interactions were shown to influence intracellular lipid synthesis, fat accumulation, oxidative stress, and hepatic inflammation. Upon exposure of hepatocytes to extracellular lipid *in vitro*, or after administering chemicals and/or diets to C57BL/6 mice to induce MASH or MASH-related fibrosis, TM4SF5 expression was associated with elevated KEAP1 levels and MASLD phenotypes, while NRF2 levels remained unchanged. Mice with a non-DNA-binding Nrf2 mutant exhibited less efficient development of MASH-related fibrosis following HFDCCl_4_ treatment, compared to interbred *Alb*-TG^Tm4sf5^×Nrf2^Mut^ mice. Additionally, *Tm4sf5*^-/-^ KO×Nrf2^Mut^ mice did not demonstrate notable MASH-related fibrosis, even with HFDCCl_4_ challenge. In liver lysates from interbred animals, diet-induced Keap1 upregulation that led to MASH-related fibrosis in *Alb*-TG^Tm4sf5^ mice was accompanied by enhanced lipid synthesis, ROS production, and hepatic inflammation, all of which were inhibited by Keap1 knockdown. Collectively, these results indicate that in TM4SF5-driven MASLD progression, TM4SF5-dependent KEAP1 regulation (notably, increased expression in response to aberrant lipid metabolism and deposition) and subsequent biphasic modulation of CD36 are central to the worsening of MASLD without requiring Nrf2 DNA-binding or transcriptional activation (Fig. 7).

As a member 5 of the transmembrane 4 L six family, TM4SF5 engages in the formation of protein-protein complexes with various membrane proteins, receptors, and transporters, as well as cytosolic proteins such as c-Src, FAK, and mTOR 27-29. These TM4SF5-associated proteins may undergo protein degradation or stabilization through intracellular trafficking or localization to lysosomes, similar to the fate of SLAMF7 immune checkpoint in NK cells 30 or by remaining at the plasma membrane such as integrin α5 31. This study demonstrated that TM4SF5 WT is capable of binding to KEAP1, which leads to the ubiquitination and proteasomal degradation of KEAP1, whereas several TM4SF5 mutants (including extracellular R113Q, N138A, C145A, and intracellular C189A) disrupted this binding. The WT TM4SF5-bound KEAP1 was ubiquitinated under basal conditions, conferring a longer half-life despite its lower abundance compared to TM4SF5-deficient cells, and the point mutation (C189A) in TM4SF5 prevented this binding-induced decrease in KEAP1 expression. Keap1 functions as a substrate adaptor protein for a Cul3-dependent E3 ubiquitin ligase complex, with being assembled into a functional E3 ubiquitin ligase complex with Cul3 and Rbx1 that targets multiple lysine residues located in the N-terminal Neh2 domain of Nrf2 for ubiquitin conjugation 32. C151S mutation in the N-terminal BTB (Broad complex, Tramtrack, and Bric-a-Brac) domain of Keap1 results in blocking of Nrf2 ubiquitination and rather increased ubiquitination of Keap1 33. Therefore, under normal and basal conditions without inflammatory oxidative stress, TM4SF5-bound Keap1 might lead to degradation of Keap1, whereas in inflammatory and oxidative stresses TM4SF5 might loss binding to Keap1 leading to its stabilization via molecular effects as BTB C151S mutation, although it should further be examined whether it is the case in next coming studies. Under basal conditions, TM4SF5-mediated reduction of KEAP1 in hepatocytes inversely correlated with the levels of CD36, a transmembrane glycoprotein involved in fatty acid uptake and lipid metabolism 34. Furthermore, upon exposure to extracellular lipids to simulate chronic pathological conditions or hyperlipidemia, TM4SF5-expressing hepatocytes exhibited increased KEAP1 expression. This proportional relationship between TM4SF5 and KEAP1 became evident in mice fed an MCD diet or HFDCCl_4_ during MASH or MASH-associated fibrosis *in vivo*. Meanwhile, the levels of CD36 expression displayed a biphasic pattern (see below), correlating with TM4SF5 expression and MASLD progression in mouse models. Nonalcoholic steatotic or MASH mouse models fed an MCD diet exhibit reduced CD36 expression, even in advanced MASLD or MASH states 26. In contrast, livers from human MASLD patients display a noteworthy association among TM4SF5, KEAP1, and CD36 expression; Compared to healthy controls, all three proteins are elevated in MASH patients (with fibrosis scores up to F3); There is a positive correlation between TM4SF5 and CD36 during early disease stages (presumably fibrosis scores up to F2), but this relationship becomes negative at later stages of chronic fibrosis or cirrhosis (i.e., F3 to F4) in those with high TM4SF5 expression (the TM4SF5^High^ group, top 50% expression), while patients in the TM4SF5^Low^ group (bottom 50% expression) do not exhibit significant differences in CD36 levels across stages of fibrosis. Therefore, the biphasic regulation of CD36 expression throughout the course of MASH requires further elucidation, particularly in the context of its potential connection to TM4SF5-mediated MASLD. It is possible that under baseline conditions, the TM4SF5-KEAP1-CD36 interaction promotes KEAP1 ubiquitination and degradation, which could stabilize CD36, facilitating regulated lipid uptake and metabolism. In settings of chronic hyperlipidemia, however, Keap1 can dissociate from the complex, resulting in prolonged KEAP1 stability in both TM4SF5-positive and -negative cells. This stabilization of KEAP1 may consequently contribute to lipid toxicity, including the formation of vacuole-like cytosolic structures, increased oxidative stress, and inflammation, ultimately culminating in MASH-associated fibrosis.

The NRF2-KEAP1 pathway acts as a central regulator of cellular defense mechanisms against environmental oxidative stressors 15. NRF2 mitigates ROS toxicity by promoting the expression of antioxidant enzymes, while KEAP1 modulates NRF2 activity through the detection of oxidative stress 16. Under basal conditions in the absence of oxidative stress, the CUL3-KEAP1 ubiquitin ligase complex targets NRF2 for ubiquitination, resulting in its proteasomal degradation 32. Notably, the present study demonstrated that the TM4SF5-KEAP1 complex could induce ubiquitination of KEAP1, even though the specific ubiquitin ligase was not identified, suggesting a potential indirect involvement of TM4SF5 in facilitating KEAP1 ubiquitination by recruiting an E3 ligase (see above). While extensive research has established an inverse correlation between NRF2 and KEAP1 that influences the inflammatory microenvironment 15, 16, 18, this study presents evidence that KEAP1 in TM4SF5-positive hepatocytes and liver tissues may be regulated distinctly in response to lipid exposure, independent of NRF2 expression levels and transcriptional activity. Support for TM4SF5-dependent effects on KEAP1 expression and function that occur independently of NRF2 was provided by findings in Nrf2*^Mut^* animals, where a defect in Nrf2 prevents binding to DNA and results in the loss of transcriptional activation of antioxidant gene programs 20. Specifically, Nrf2^Mut^ mice exhibited hepatocellular injury but with reduced fat droplet accumulation following HFDCCl_4_ administration compared with WT controls. Interbred *Alb*-TG^Tm4sf5^×Nrf2^Mut^ mice exhibited markedly severe hepatocellular injury, lipid accumulation, and inflammation, whereas *Tm4sf5*^-/-^ KO×Nrf2^Mut^ mice did not display these features. Thus, when TM4SF5 is highly expressed in hepatocytes, NRF2-KEAP1 signaling may be altered such that KEAP1 potentially regulates hepatic ROS independently of NRF2. In contrast, even with Keap1 suppression in *Alb*-TG^Tm4sf5^ mice maintained on an MCD diet, MASLD characteristics were suppressed compared to animals without Keap1 suppression, suggesting that the Tm4sf5-Keap1 interaction could be essential for MASLD progression. As shown in this study that Keap1 could modulate cellular ROS levels independent of Nrf2, PGAM5 (mitochondrial serine/threonine protein phosphatase)-PINK1-Parkin pathway can function in ROS sensing, irrelevantly to Nrf2 35. Keap1 binds PGAM5 and causes proteosomal degradation of PGAM5 in a basal condition, meanwhile, in oxidative stress, Keap1 is dissociated from PGAM5, leading to PGAM5 accumulation that interferes with PINK1 processing (for PINK1 stabilization) and Parkin translocation to mitochondria, eventually resulting in removal of ROS and ROS-producing mitochondria. Therefore, in the basal condition where TM4SF5 expression led to a decrease in Keap1, an increased activation of PINK1-Parkin pathway may lead to removal of ROS. Meanwhile, in oxidative stress condition following abnormal lipid accumulation in liver, TM4SF5-mediated increase in Keap1 might cause more degradation of PGAM5 not leading to activation of PINK1-Parkin pathway in development of inflammatory ROS accumulation for MASH. However, the linkage TM4SF5-KEAP1-PGAM5 remains to be explored. Nevertheless, the pharmacological targeting of the TM4SF5-KEAP1 interaction could serve as a promising therapeutic approach for MASH-associated fibrosis 12, 36.

We observed that TM4SF5-mediated upregulation of KEAP1 following lipid treatment was inversely correlated with CD36 expression level. Analysis of GSE135251 demonstrated that *CD36* mRNA expression is elevated in fatty liver (or NAFL) and MASH patients across F0~1 to F3 stages, whereas this increase was not observed in the F4 group, when compared to control individuals. CD36 deficiency in mice is found to attenuate HFD-induced hepatic steatosis while exacerbating MCD diet-induced steatohepatitis 26, and that the expression of CD36 in hepatocytes was weak, whereas under surplus lipids the expression of CD36 was significantly upregulated 37. Further notably, *CD36* mRNA levels in human *TM4SF5*^High^ MASLD patients within GSE135251 were increased in those with F1 to F2 fibrosis scores but declined or decreased at F3 to F4 stages; such changes were not significant in the *TM4SF5*^Low^ group. Additional analysis of 52 morbidly obese patients [body mass index (BMI): 53.82 ± 1.41; age: 45 ± 10.50; 15 males/37 females] revealed associations between CD36 and both apoptosis mediators and MASH- and fibrosis-related histological features, indicating that apoptosis levels positively correlate with CD36 expression 38. These findings support the possibility that, in cases with advanced fibrosis such as F3 ~ F4, removal of apoptotic cells along with increased hepatocyte regeneration may lead to reduced CD36 expression. In murine macrophages exposed to oxidatively modified LDL (oxLDL), CD36 may act as both an upstream and downstream effector of Nrf2 39. In parallel, Keap1-KO mice subjected to a HFD exhibit increased features of metabolic syndrome, including greater body weight, expansion of white adipose tissue, inflammation, and upregulation of lipogenic genes 40. This earlier study of Keap1-KO supports the concept that Keap1 downregulation may be induced by TM4SF5 overexpression in *Alb*-TG^Tm4sf5^ for MASH-associated fibrosis. Moreover, loss of CD36 reduces ROS production in response to FeCl_3_-mediated vascular injury in a mouse carotid artery thrombosis model 41. Therefore, TM4SF5 and CD36 may exhibit a biphasic relationship under conditions of chronically abnormal lipid metabolism, as demonstrated both in the current study and in GSE135251, and such a biphasic trend in *CD36* mRNA changes in MASLD or MASH patients depending on fibrotic scores was also in the case of GSE48452. Notably, NRF2 activation, accompanied by KEAP1 downregulation, has been shown to augment CD36 expression and promote intracellular lipid accumulation in mouse insulinoma MIN6 cells 42 and in animals with HFD-induced obesity 43. Meanwhile, hepatocyte-specific CD36 knockout promotes Notch signaling and liver fibrosis in mice fed with a NASH diet, and that Notch receptor expression can be differential depending on the steatotic and steatohepatitic diets 44. Thus, it remains to examine if TM4SF5 can mimic Notch receptor. In the present study utilizing hepatocytes, Nrf2 DNA binding and thereby its transcriptional function did not appear to be essential for Tm4sf5-driven modulation of Keap1 and CD36 or for exacerbation of MASLD phenotypes, suggesting that KEAP1 downregulation could indirectly facilitate CD36 expression or stabilization; however, this mechanism warrants further investigation. In our MASH-associated fibrosis model, enhanced TM4SF5-mediated KEAP1 expression corresponded with elevated levels of lipogenic (e.g., Cd36 and Dgat1/2), inflammatory, and fibrotic markers in *Alb*-TG^Tm4sf5^ mice fed an MCD diet, compared to WT or KO counterparts. Suppression of Keap1 counteracted these increases, although the expression patterns of Nrf2 and its downstream targets Ho-1 and Nqo1 differed from those observed for Keap1. Thus, it remains possible that persistent TM4SF5 overexpression in hepatocytes enables TM4SF5-KEAP1 interactions to functionally substitute for, or bypass, components of the NRF2-KEAP1 pathway at least in part.

TM4SF5, a member of the tetraspan(in) family, serves as a signaling hub by assembling a protein-protein complex known as the TM4SF5-enriched microdomain (T_5_ERM) 45, which is analogous to structures such as the tetraspanin web, focal adhesion sites, or caveolae 27. Although NRF2 and KEAP1 are primarily cytosolic proteins, the NRF2-KEAP1 complex or unbound KEAP1 may localize near membrane subdomains such as T_5_ERMs. Because TM4SF5 can shuttle between the plasma membrane, lysosome, lysosome-mitochondria contact sites, and even extracellular small vesicles 19, 28, 31, 45, TM4SF5-Keap1 complexes could interact with cytosolic proteasomes for degradation at endosomal membranes or form a triple complex with CD36 at the plasma membrane, facilitating the uptake of extracellular lipids or fatty acids. Recently, TM4SF5-specific isoxazole compounds or anti-TM4SF5 monoclonal antibody (clone S43) have been used therapeutically in animal models of HCC or PDX 30, 46. These agents are capable of inhibiting the function of hepatocyte TM4SF5 by impeding intracellular signaling and disrupting protein-protein interactions. In our current study, TSAHC [4'-(*p*-toluenesulfonylamido)-4-hydroxychalcone], another TM4SF5-specific chalcone compound 22, was found to block TM4SF5-mediated KEAP1 downregulation, inhibit the binding between TM4SF5 and KEAP1, and mitigate HFDCCl_4_-induced MASLD in *Alb*-TG^Tm4sf5^ mice. Therefore, isoxazole or S43 mAb may also hold therapeutic potential for MASLD or MASH-associated fibrosis. Indeed, we have obtained preliminary data supporting the therapeutic efficacy of the TM4SF5-specific isoxazole compound for diet-induced MASH in an ongoing project.

In summary, we demonstrate that hepatocyte TM4SF5 is capable of stabilizing KEAP1, resulting in increased intracellular lipid accumulation following lipid treatment, whereas under basal conditions, TM4SF5 facilitates the downregulation of KEAP1. TM4SF5 under lipid exposure may promote oxidative stress and inflammation, thereby contributing to MASLD progression in mice subjected to stressful diets. These TM4SF5-mediated effects were not dependent on NRF2 levels or its transcriptional function. Consequently, the TM4SF5 and KEAP1 linkage may represent a novel potential therapeutic target for MASLD.

## Supplementary Material

Supplementary figures.

## Figures and Tables

**Table 1:** The target sequences of siRNAs.

	Target				Sequence (5′→ 3′)
*Homo sapiens*	TM4SF5	siRNA	#2	Sense	ACC AUG UGU ACG GGA AAA UGU GC
				Anti-Sense	GCA CAU UUU CCC GUA CAC AUG GU
			#4	Sense	CCA UCU CAG CUU GCA AGU C
				Anti-Sense	GAC UUG CAA GCU GAG AUG G
			#7	Sense	CCU CCU GCU GGU ACC UAA U
				Anti-Sense	AUU AGG UAC CAG CAG GAG G
			#8	Sense	GCU UGC AAG UCU GGC UCA U
				Anti-Sense	AUG AGC CAG ACU UGC AAG C
	KEAP1	siRNA	#1	Sense	GGC CUU UGG CAU CAU GAA CUU
				Anti-Sense	GUU CAU GAU GCC AAA GGC CUU
			#2	Sense	GGA CAG UUA UUU UGU UGA UAA GUA A
				Anti-Sense	UUA CUU AUC AAC AAA AUA ACU GUC CAU
	NRF2	siRNA	#1	Sense	AAG AGU AUG AGC UGG AAA AAC TT
				Anti-Sense	GUU UUU CCA GCU CAU ACU CU UTT
			#2	Sense	GGG AGG GAG CUA UUA UCC AUU U
				Anti-Sense	AUG GAU AAU AGC UCC UCC CUU

**Table 2:** The sequences of primers for PCR

Species	Gene	Sequence (5'-3')
Homo sapiens	GAPDH-F	GGT GTG AAC CAT GAG AAG TAT GA
	GAPDH-R	GAG TCC TTC CAC GAT ACC AAA G
	TM4SF5-F	CTT GCT CAA CCG CAC TCT AT
	TM4SF5-R	ATC CCA CAC AGT ACT ATC TCC A
	KEAP1-F	CAC AAC AGT GTG GAG AGG TAT G
	KEAP1-R	CGG CAT AAA GGA GAC GAT TGA
	NRF2-F	GTT GCC CAC ATT CCC AAA TC
	NRF2-R	CGT AGC CGA AGA AAC CTC AT
	HO1-F	ACC AAG TTC AAG CAG CTC TAC
	HO1-R	GCA GTC TTG GCC TCT TCT ATC
	CHOP-F	CAA GAG AGG GTC TTG GAG AAA G
	CHOP-R	GCC TGC CAG AAG TCA TGT AT
	CAT-F	CTG GGA GAC GAG ACA CAT AAA C
	CAT-R	TGG TCA CTC CCT CTA CAT TCT
	SOD1-F	GTG CAG GGC ATC ATC AAT TTC
	SOD1-R	GGC CTT CAG TCA GTC CTT TAA T
	ATF4-F	CCC TTC ACC TTC TTA CAA CCT C
	ATF4-R	TTC ACT GCC CAG CTC TAA AC
	PERK-F	GGA AAC GAG AGC CGG ATT TAT
	PERK-R	TAT GGC AGC TTC CTG TTC TTC
	IKKα-F	CCG AAA GCT GCT CAA CAA AC
	IKKα-R	TCG AAT CCC AGA CCC TAT ATC A
	IKKβ-F	CAG AAT CAT CCA TCG GGA TCT AA
	IKKβ-R	ATC CAG CTC CTT GGC ATA TC
	TNFα-F	CCA GGG ACC TCT CTC TAA TCA
	TNFα-R	TCA GCT TGA GGG TTT GCT AC
	TGFβ-F	GCC GAA TTC CGG ATC TAC AA
	TGFβ-R	CTC CTG GAG CAC CTG ATA AAC
	CCL2-F	TCA TAG CAG CCA CCT TCA TTC
	CCL2-R	CTC TGC ACT GAG ATC TTC CTA TTG
	CCL3-F	CCC TAG TCT CCA GGT ATG AGA A
	CCL3-R	GGA TTC TGC CTC TTG CTA ACT
	CCL4-F	CTC ATG CTA GTA GCT GCC TTC
	CCL4-R	GGC TGC TGG TCT CAT AGT AAT C
	CCL5-F	TGC CCA CAT CAA GGA GTA TTT
	CCL5-R	GAT GTA CTC CCG AAC CCA TTT
	CCL13-F	GCA CTC AAC GTC CCA TCT AC
	CCL13-R	GGT GGT GAT CAC ATA GCT CTT C
	CCL19-F	GAC TGT CTC TGC AAG CTC TAT T
	CCL19-R	GGC TTA GTG TCT GCC ATT CT
	CCL20-F	ACC ATG TGC TGT ACC AAG AG
	CCL20-R	TGT ATC CAA GAC AGC AGT CAA A
	CCL21-F	CAG CCA CAC AAA GAA ACA AAG A
	CCL21-R	TCC CAA AGT GCT GGG ATT AC
	CCL22-F	GCT GTG GCA TCT AGG GTA TTT
	CCL22-R	GTC CTG GTT CAC CAT CCA TT
	CXCL1-F	ACT CAA GAA TGG GCG GAA AG
	CXCL1-R	CCC TTC TGG TCA GTT GGA TTT
	CXCL2-F	GCA TCG CCC ATG GTT AAG A
	CXCL2-R	TCA GGA ACA GCC ACC AAT AAG
	CXCL3-F	TCA CCT CAA GAA CAT CCA AAG T
	CXCL3-R	AGA CAA GCT TTC TTC CCA TTC T
	CXCL5-F	CAA TCT TCG CTC CTC CAA TCT C
	CXCL5-R	AGG AGG CTC ATA GTG GTC AA
	CXCL6-F	CCC TGG ACC CAG TAA GAA TAA G
	CXCL6-R	TAA ACT TCA GGG AGA AGC AGC GTA G
	CXCL8-F	CTT GGC AGC CTT CCT GAT TT
	CXCL8-R	GGG TGG AAA GGT TTG GAG TAT G
	CXCL10-F	GTA ATA ACT CTA CCC TGG CAC TAT AA
	CXCL10-R	GAT GGG AAA GGT GAG GGA AAT A
	CXCL12-F	ATG CCC ATG CCG ATT CTT
	CXCL12-R	CAC ACT TGT CTG TTG TTG TTC TT
	CXCL16-F	GCG TCA CTG GAA GTT GTT ATT G
	CXCL16-R	TGG TAA GCT CTC AGG TGT TTC
Mus musculus	Gapdh-F	GTG GCA AAG TGG AGA TTG TTG
	Gapdh-R	CGT TGA ATT TGC CGT GAG TG
	Tm4sf5-F	CGA ATT GGA CCC AAA TGC TTA AT
	Tm4sf5-R	CGC CTC ACA CAA ATT CCA AAG
	Ho1-F	ACA TCG ACA GCC CCA CCA AGT TCA A
	Ho1-R	CTG ACG AAG TGA CGC CAT CTG TGA G
	Nqo1-F	CTC GAA TCT GAC CTC TAT GCT ATG
	Nqo1-R	GAT GAC TCG GAA GGA TAC TGA AA
	Sod1-F	CGG ATG AAG AGA GGC ATG TT
	Sod1-R	GAG AGT GAG ATC ACA CGA TGT T
	Atf4-F	CCA CTC CAG AGC ATT CCT TTA G
	Atf4-R	CTC CTT TAC ACA TGG AGG GAT TAG
	IKKα-F	GGA CCA TGC GAA TGT TGT AAA G
	IKKα-R	CCT CCA GAA CAG TAC TCC ATT G
	IKKβ-F	TGA GAA GAA AGT TCG GGT GAT T
	IKKβ-R	CCT CGT TCA TAA GGC TCA CTA C
	IFNγ-F	CTC TTC CTC ATG GCT GTT TCT
	IFNγ-R	TTC TTC CAC ATC TAT GCC ACT T
	Adgre1-F	TAC CAC TTG CCC AGC TTA TG
	Adgre1-R	GGG CCT TGA AAG TTG TTG GTT TG
	Csf1r-F	CCT ACC CTA GCA TAC AGC ATT AC
	Csf1r-R	ATG GCC CTT TGG GTG ATA AA
	Ccl2-F	CTC ACC TGC TGC TAC TCA TTC
	Ccl2-R	ACT ACA GCT TCT TTG GGA CAC
	Ccl5-F	CTG CTG CTT TGC CTA CCT
	Ccl5-R	TCG AGT GAC AAA CAC GAC TG
	Ccl20-F	TGA ACC TCC TCA GCC TAA GA
	Ccl20-R	CCC AGC TGT GAT CAT TTC CT
	Cxcl1-F	GCT GGG ATT CAC CTC AAG AA
	Cxcl1-R	TGG CTA TGA CTT CGG TTT GG
	Cxcl3-F	CAC CCT ACC AAG GGT TGA TTT
	Cxcl3-R	CAT CCT TGA GAG TGG CTA TGA C
	Cxcl5-F	TGA ACT CCC TGC TTT GAT GAG
	Cxcl5-R	CCG ATA GTG TGA CAG ATA GGA AAG
	Cxcl10-F	TCC TAA TTG CCC TTG GTC TTC
	Cxcl10-R	CAT GGC TTG ACC ATC ATC CT
	Cxcl15-F	CAC TCA AGA GCT ACG ATG TCT
	Cxcl15-R	GTT GCA GTA AAT GGT CTC GAA TAT C

**Table 3:** The primary antibodies used in the study.

Primary antibodies	Source.	Cat.
C-terminal TM4SF5 (CGG-190RKKQDTPH197)	Abclon, Seoul, Korea	-
EC2 TM4SF5 (aa 118-130)	Pro-Sci, Poway, CA	-
α-Tubulin	Santa Cruz Biotechnology, Inc.	sc-2053
β-Actin (C4)	Santa Cruz Biotechnology, Inc.	sc-47778
HA	Cell signaling Technology	#3724
	Biolegend	901515
StrepMAB-Classic, HRP conjugate	IBA Lifesciences	2-1509-001
FLAG	Cell signaling Technology	#2368
	Sigma-Aldrich	F1804
	Novus Biologicals	NB600-344
KEAP1	Sigma-Aldrich	K2769
	Novus Biologicals	NBP2-03319
NRF2	Novus Biologicals	NBP1-32822
HO-1	Invitrogen	#MA1-112
NQO1	Invitrogen	#PA5-19624
FASN	Cell signaling Technology	#3189
DGAT1	Santa Cruz Biotechnology, Inc.	sc-271934
DGAT2	Santa Cruz Biotechnology, Inc.	sc-293211
SQSTM1/p62	Cell signaling Technology	#5114
LC3B	Cell signaling Technology	#2775
CAT	Santa Cruz Biotechnology, Inc.	sc-271803
SOD1	Cell signaling Technology	#37385
TNF-α	Abcam	ab1793
TGF-β	Invitrogen	#MA5-23795
F4/80	Cell signaling Technology	#70076
MCP-1/CCL2	Invitrogen	#MA5-17040
RANTES/CCL5	Santa Cruz Biotechnology, Inc.	sc-514019
MIP-3α/CCL20	Santa Cruz Biotechnology, Inc.	sc-517441
IP-10/CXCL10	Santa Cruz Biotechnology, Inc.	sc-101500

## Abbreviations

*Alb*-TG^Tm4sf5-Flag^: transgenic C57BL/6 mice overexpressing hepatocyte-specific Flag-tagged Tm4sf5
HCC: hepatocellular carcinoma
HFDCCl_4_: treatment for MASH mice model with high fat diet (60% kcal fat) and intraperitoneal injections of carbon tetrachloride (5 mg/kg, twice weekly) over a 12-week period
Huh7_TM4SF5-KO_ cells: Huh7 cells with genetic knockout of *TM4SF5*
KEAP1: Kelch-like ECH-associated protein 1
MASH: Metabolic dysfunction-associated steatohepatitis
MASLD: metabolic dysfunction-associated steatotic liver disease
NRF2: transcription factor NF-E2-related factor 2
Nrf2^Mut^: C57BL/6 mice with Nrf2 mutant incapable of binding DNA due to a deletion in the CNC bZIP region (amino residues 452 to 560)
PA: palmitic acid
ROS: reactive oxygen species
TM4SF5: Transmembrane 4 L six family member 5
