# Supplementary material for: TM4SF5-mediated KEAP1 Regulation in Hepatocytes Irrelevant to NRF2 Expression and Activity Promotes Oxidative Stress and Inflammation to Develop Metabolic Dysfunction-Associated Steatotic Liver Disease
Source: Int J Biol Sci. 2026 Jan 1;22(3):1103–25. doi: 10.7150/ijbs.126251 (PMC12836525; doi:10.7150/ijbs.126251)
Supplement: Supplementary file 1 — Supplementary figures. [file ijbsv22p1103s1.pdf]

TM4SF5-mediated KEAP1 Regulation in Hepatocytes Irrelevant to NRF2 Expression and Activity Promotes Oxidative Stress and Inflammation to Develop Metabolic Dysfunction-Associated Steatotic Liver Disease

Eun-Ae Shin[1,2], Haesong Lee[1,2], Kyung-hee Pyo[1,2], Wonsik Kim[1,2], Soyeon Kim[1,2], Jae-Ho Lee[1,2], Seo Hee Jin[1,2], Eunmi Kim[2], Soo-Min Byeon[1,2], Dong Joo Kim[1,2], Young Jun Cho[1,2], Tae Won Kim[1,2], Minjae Ohn[1,2], Hyojung Lee[1,2], Jeongwon Lee[1,2], Jinwook Jeong[1], Doojin Kim[3], Jie Zheng[1,2], Han Ah Lee[4], Hwi Young Kim[5,#], Young-Joon Surh[1,2], and Jung Weon Lee[1,2,*].

Supplementary Figures S1 to S5

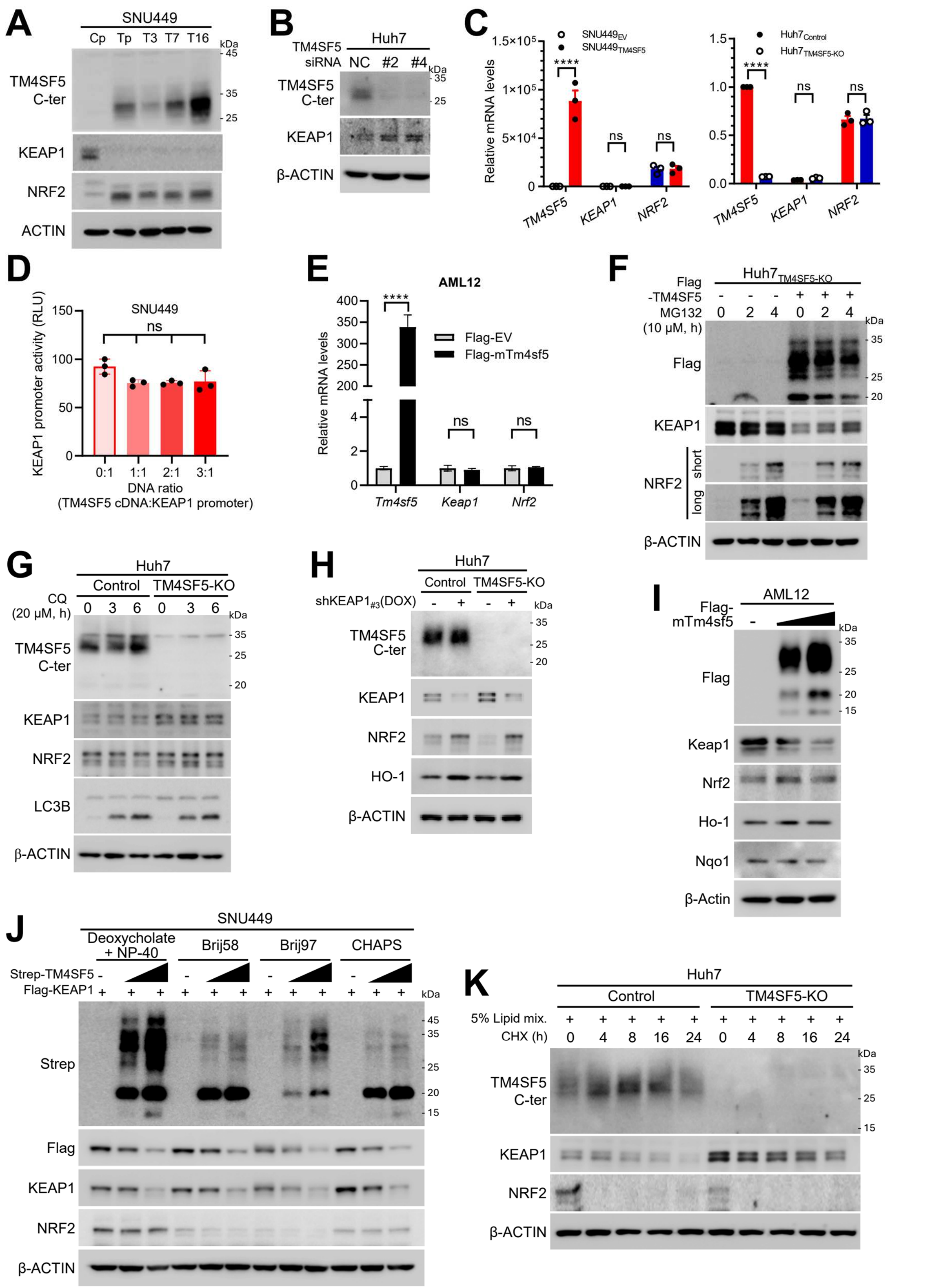

Supplementary Figure S1. TM4SF5-mediated downregulation of KEAP1 under basal conditions. (A-B) Stable SNU449 cell clones (Cp, pooled clone for control vector; Tp, pooled

clone for exogenous TM4SF5 expression; T3, T7, or T16 as single cell clones for TM4SF5 expression) (A) or Huh7 cells transfected with non-specific siRNA (NS) or TM4SF5-targeting siRNAs (#2 and #4, Table 1) were collected for immunoblot analysis. **(C)** Subconfluent hepatocytes stably transfected with the indicated vectors were subjected to qRT-PCR for the assessment of target gene expression. **(D)** SNU449 cells were cotransfected with TM4SF5 cDNA and KEAP1 promoter construct at specified ratios for 24 h, and KEAP1 promoter activity was subsequently measured. **(E)** Murine AML12 hepatocytes stably expressing either empty vector (EV) or mouse Tm4sf5 were analyzed by qRT-PCR for the specified transcripts. *, $P < 0.05$; **, $P < 0.01$; ***, $P < 0.001$; ***, $P < 0.0001$; ns, not significant, one-way ANOVA. **(F-H)** $Huh7_{Control}$ or $Huh7_{TM4SF5\text{-}KO}$ cells were treated with MG132 **(F)**, chloroquine (CQ, **G**), or doxycycline (DOX, **H**) at the indicated concentrations and durations before cell harvest. **(I-J)** Subconfluent AML12 or SNU449 hepatocytes were stably (I) or transiently (J) transfected with the specified cDNAs for 48 h, followed by preparation of whole cell extracts using lysis buffers containing Triton X-100 (I) or alternative detergents (J) and analyzed by immunoblotting. **(K)** Huh7 cell variants were exposed to 5% lipid mixture for 24 h, followed by treatment with cycloheximide (CHX). Whole cell extracts were collected at different intervals after CHX exposure for immunoblot analysis. Data represent results from three independent experiments. See also Fig. 1.

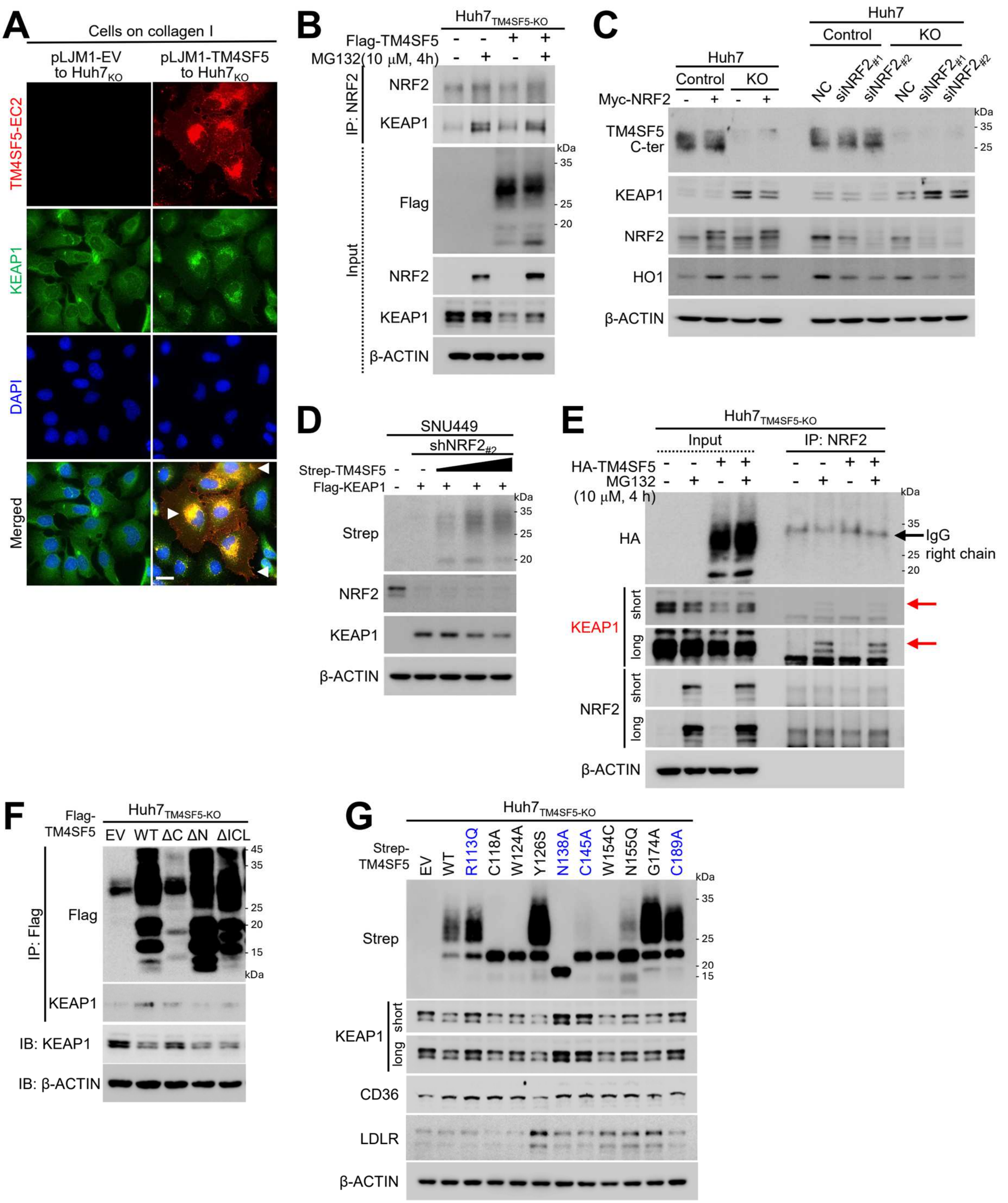

Supplementary Figure S2. TM4SF5 interacts with KEAP1, but not with NRF2, mediating for TM4SF5-mediated KEAP1 downregulation. **(A)** Huh7 cells stably transfected with the indicated cDNAs were replated onto collagen I-precoated slide glasses prior to immunostaining and imaging. Scale bar: 100 μm. **(B)** Subconfluent hepatocytes with either stable or transient transfection of the indicated cDNAs, treated with or without MG132 (D) for 4 h, were harvested for whole cell extracts using a Triton X-containing lysis buffer. Normalized extracts underwent (immuno)precipitation using anti-NRF2, followed by immunoblotting. **(C-E)** Subconfluent

hepatocyte variants that received stable (C) or transient (D) transfections with the indicated cDNAs (C-E), siRNAs (C), or shRNAs (D) targeting either non-specific sequences (NS) or NRF2 sequences (#1 or #2, Table 1), were collected for whole cell lysate (Input) preparation. For (E), cells were treated with MG132 (10 μM, 4 h) prior to extract preparation. The normalized whole cell lysates were subjected to immunoblot analysis. **(F and G)** Subconfluent $Huh7_{TM4SF5\text{-}KO}$ hepatocytes transiently transfected with TM4SF5 mutants, either deletion mutants (F) or point mutants (G), were subjected to (immuno) precipitation using anti-Flag, followed by immunoblotting for the indicated molecules. Mutants highlighted in blue did not display decreased KEAP1 levels, in contrast to TM4SF5 wildtype (WT). The data represent three independent experiments. See also Fig. 1.

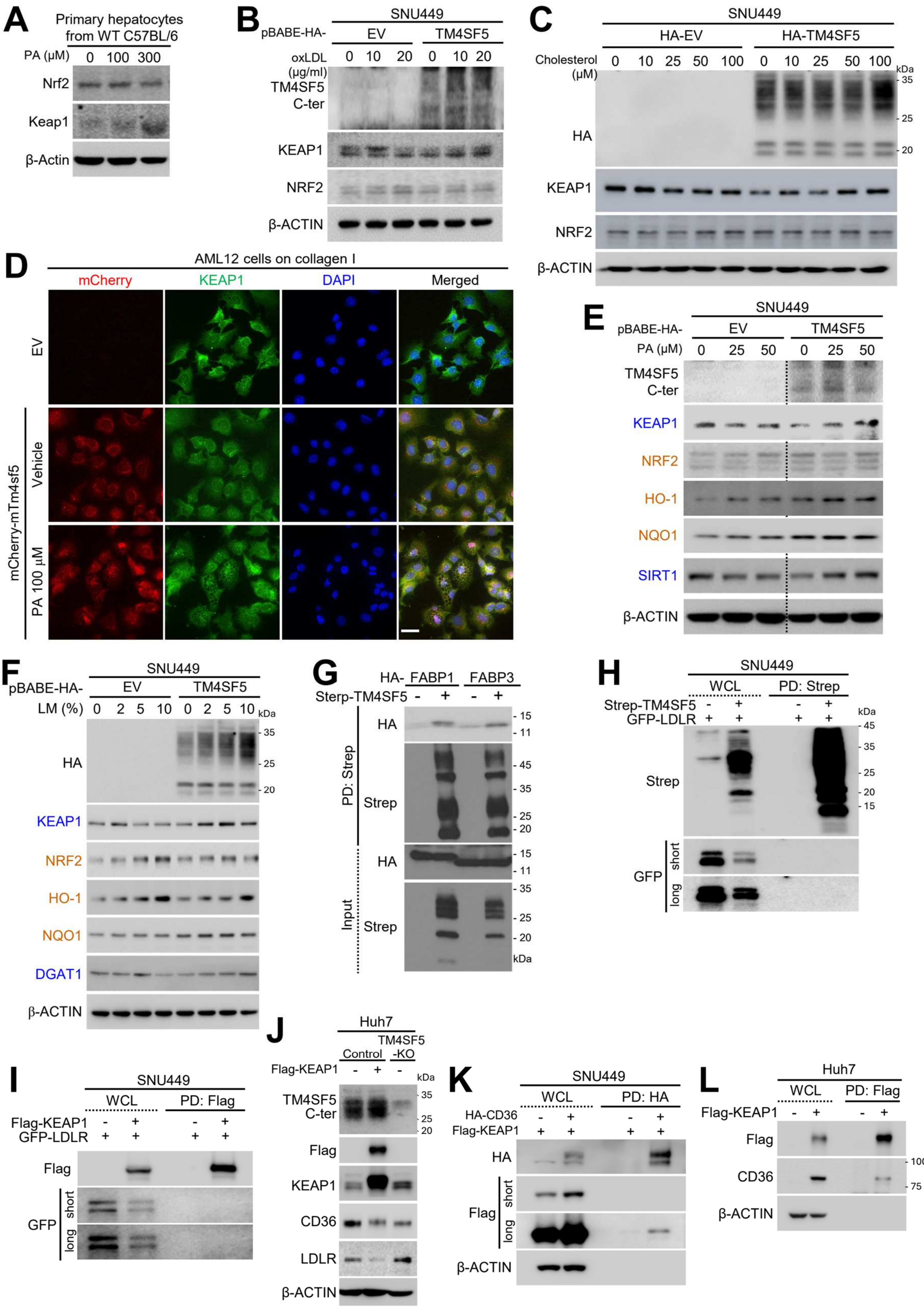

Supplementary Figure S3. TM4SF5-mediated upregulation of KEAP1 upon stressful

lipid-induced cellular stress. Primary hepatocytes isolated from WT C57BL/6 male mice (A), or subconfluent hepatocytes subjected to stable or transient transfection with the indicated cDNAs, were treated with PA (A), oxidized LDL (oxLDL, B), or cholesterol (C) at the specified concentrations for 24 h prior to preparation of whole cell extracts and subsequent immunoblot analysis. (D) Subconfluent normal murine AML12 hepatocytes, stably expressing either empty vector (EV) or mCherry-Tm4sf5, were replated onto collagen I-precoated slide glasses for subsequent immunostaining and imaging. Scale bar: 100 μm. (E and F) Subconfluent SNU449 hepatocytes, either untransfected or stably expressing TM4SF5, were exposed to PA (E) or LM (F) at indicated concentrations for 24 h and subsequently harvested to prepare whole cell lysates using lysis buffer containing Triton X-100. All lysates were normalized and subjected to immunoblotting for the detection of specified proteins. **(G-I)** SNU449 cells not treated with lipids were collected, normalized, and used for coimmunoprecipitation with either streptavidin-agarose (G and H) or anti-Flag antibody (I), followed by immunoblotting for the designated molecules. **(J-L)** Subconfluent hepatocytes harboring stable knockout (KO) or expressing the indicated cDNAs by stable or transient transfection were collected, and whole cell lysates (WCL) were prepared using Triton X-100-containing buffer, normalized, and used for immunoblotting (J) or for immunoprecipitation employing anti-HA (K) or Flag (L) antibodies prior to immunoblot analysis of the indicated molecules. Data are representative of three independent experiments. See also Fig. 2.

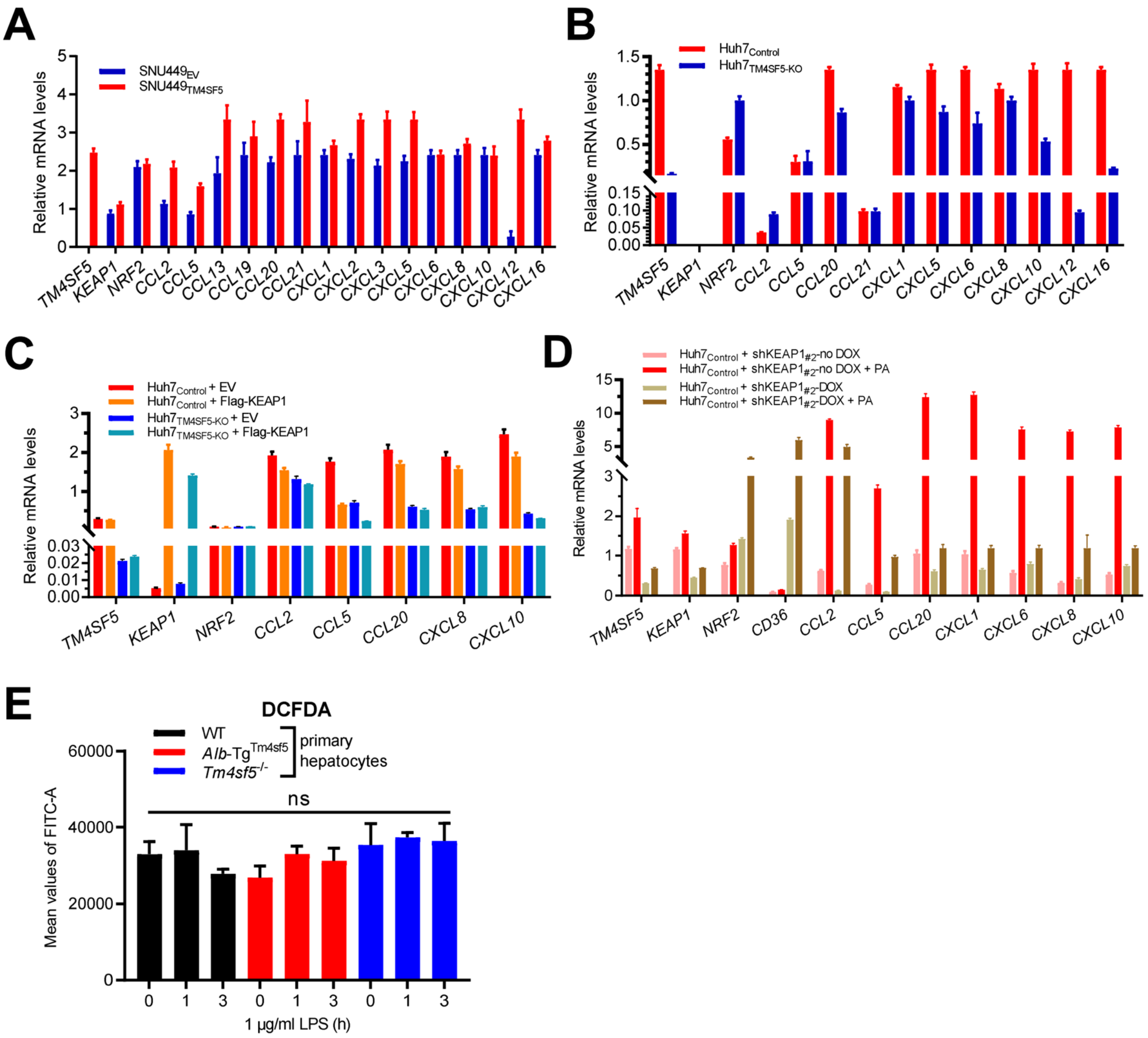

Supplementary Figure S4. TM4SF5-mediated upregulation of cytokines and chemokines. **(A)** Subconfluent SNU449 hepatocytes stably expressing either empty vector (SNU449$_{EV}$) or TM4SF5 (SNU449$_{TM4SF5}$) were collected, followed by qRT-PCR analysis to assess the expression levels of the indicated molecules. **(B-C)** Subconfluent Huh7$_{Control}$ cells (parental, endogenously TM4SF5-expressing) or Huh7$_{TM4SF5\text{-}KO}$ cells, either without (B) or with transient transfection of empty vector (EV) or Flag-KEAP1 cDNAs (C), were harvested prior to qRT-PCR for the detection of the indicated molecules. **(D)** Subconfluent Huh7$_{Control}$ cells were treated with or without (-/+) DOX to suppress KEAP1 expression (shKEAP1$_{\#2}$, Table 1) for 24 h, followed by PA treatment (100 μM) for 4 h, and then harvested for qRT-PCR analysis of the indicated targets. **(E)** Primary hepatocytes were isolated from WT, *Alb*-TG$^{Tm4sf5\text{-}Flag}$, or *Tm4sf5*$^{-/-}$ KO C57BL/6 male mice (n=4, 8-week-old) fed a NCD and subsequently treated with LPS (1 μg/ml) for 1 h to induce ROS, followed by DCFDA staining and measurement by flow cytometry. ns depicts non-significance in one-way ANOVA analysis. Data represent results from three independent experiments. See also Figs. 4 and 5.

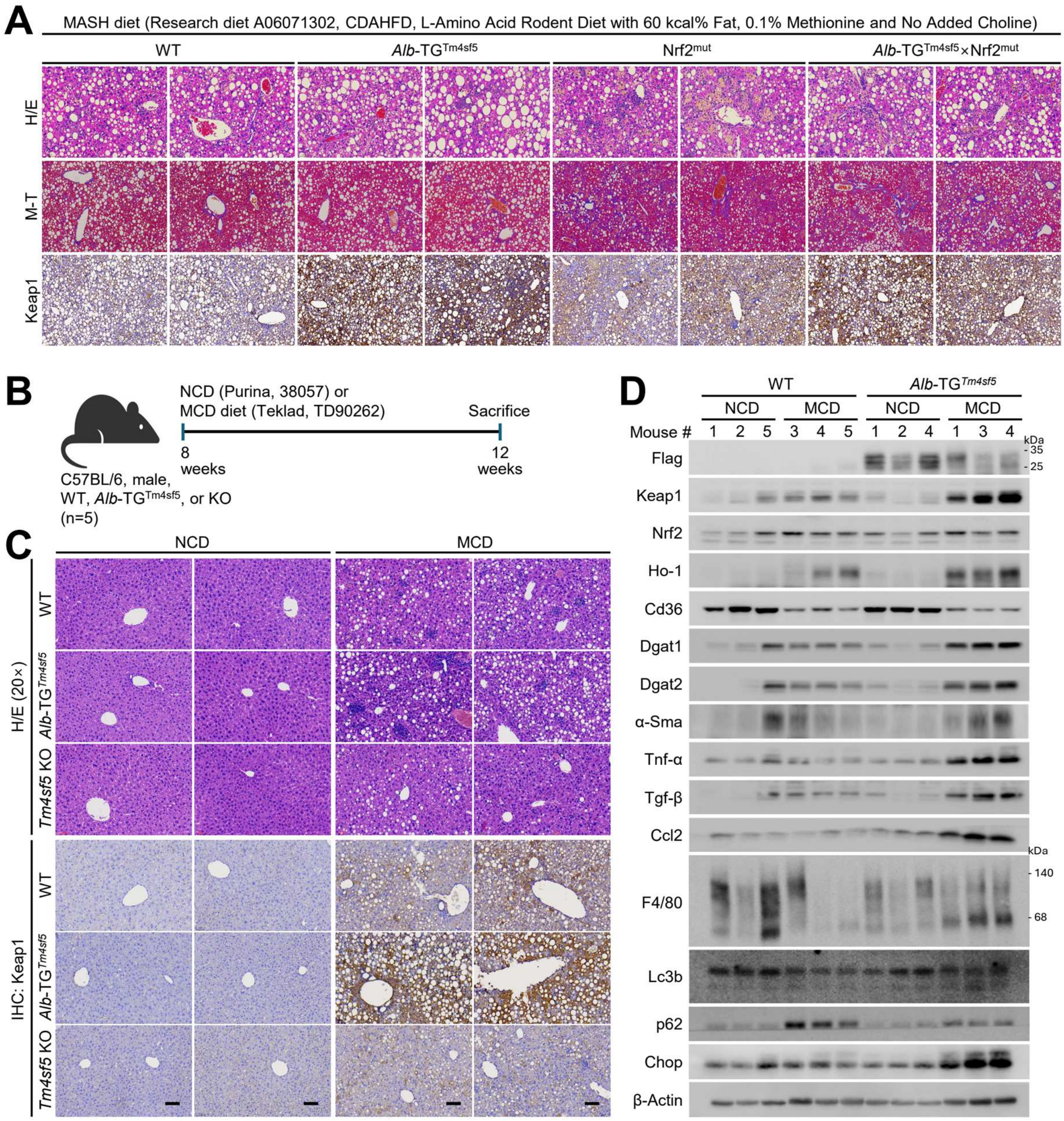

Supplementary Figure S5. TM4SF5-mediated upregulation of Keap1 during MASH-associated fibrosis occurs independently of Nrf2 DNA-binding activity. (A) Livers from WT, *Alb*-TG$^{\text{Tm4sf5-Flag}}$, Nrf2^{Mut}, and *Alb*-TG$^{\text{Tm4sf5-Flag}}$×Nrf2^{Mut} C57BL/6 male mice (n=5, 8-week-old) were maintained on the MASH diet (CDAHFD, L-amino acid rodent diet with 60 kcal% fat, 0.1% methionine and no added choline) for 12 weeks prior to processing for H&E, Masson's trichrome staining, and immunohistochemical detection of Keap1. Scale bar: 100 μm. **(B-D)** WT, *Alb*-TG$^{\text{Tm4sf5-Flag}}$, and *Tm4sf5*$^{-/-}$ KO C57BL/6 male mice (n=5, 8-week-old) were fed NCD or MCD diet for 4 weeks (B), after which livers were collected for analysis by H&E staining, immunohistochemistry (C), or immunoblotting (D). Scale bar: 100 μm. See also Fig. 6.
